# Sodium butyrate improves porcine host defense peptide expression and relieves the inflammatory response upon toll-like receptor 2 activation and histone deacetylase inhibition in porcine kidney cells

**DOI:** 10.18632/oncotarget.15714

**Published:** 2017-02-24

**Authors:** Xiujing Dou, Junlan Han, Wentao Song, Na Dong, Xinyao Xu, Wei Zhang, Anshan Shan

**Affiliations:** ^1^ Institute of Animal Nutrition, Northeast Agricultural University, Harbin 150030, P.R. China

**Keywords:** porcine kidney cells, sodium butyrate, host defense peptides, TLR2, mechanism

## Abstract

Host defense peptides (HDPs) are an important component of the innate immune system and possess direct antimicrobial and immunomodulatory activities. Dietary regulation of HDPs synthesis has emerged as a novel non-antibiotic approach to combat pathogen infection. There are species- and tissue-dependent characteristics of the regulation and mechanism of HDPs. In this study, we investigated whether the histone deacetylase inhibitor (HDACi) sodium butyrate (NaB) could induce HDP expression and the mechanism underlying NaB-regulated HDP expression in PK-15 cells. Our results revealed that NaB augmented HDP expression in PK-15 cells, including porcine β-defensin 3 (pBD3), epididymis protein 2 splicing variant C (pEP2C), pBD128, pBD123, and pBD115, but no inflammatory response occurred. Inhibition of HDAC activity was not sufficient to induce the expression of pBD3 and pEP2C in comparisons of NaB and another HDACi, trichostatin A (TSA). Concomitantly, NF-κB activation was involved in the induction of HDP expression by NaB. MAPK pathway inhibition also prevented pBD3 and pEP2C induction by NaB. Furthermore, NaB could still promote pBD3 and pEP2C expression and inhibit IL-6 production in the presence of the toll-like receptor 2 (TLR2) ligand peptidoglycan. Moreover, TLR2 could be activated by both NaB and peptidoglycan, and blocking TLR2 expression suppressed HDP induction. Finally, we further showed that increased pBD3 could decrease cytokine interleukin-18 (IL-18) and increase porcine claudin 15 (pCLDN15) contents, suggesting an immunoregulatory function of pBD3. In conclusion, this work paves the way for using HDACi-NaB to induce porcine kidney defense peptides while limiting the deleterious risk of an inflammatory response.

## INTRODUCTION

Antibiotics are the mainstay of preventive treatment and therapy for all cases of pathogenic bacterial infection, and their use has grown in the past several decades. However, the excessive use of antibiotics has accelerated the emergence of multidrug-resistant microbes. The superbug, apocalypse pig, occurs constantly, and antibiotic resistance has already been listed as one of the thirty-one global risks related to social stability in the Global Risks 2014 report. Thus, there is a need to seek new and safe alternatives to conventional antibiotics in the near future [1–3].

Host defense peptides (HDPs), which function as a sentinel or guardian in the host innate immune response, not only modulate immunity and immune-cell function under physiological conditions to treat microbial infections, but they also show direct activity against multiple pathogenic microorganisms via their unique ability to disrupt cell membranes; thus, they are also called antimicrobial peptides (AMPs) or natural “peptide antibiotics” [4]. In the kidney, epithelial cells produce and secrete AMPs [5]. There are two major classes of AMPs in mammals (humans): defensins and cathelicidins. Defensins are widely distributed in mammalian epithelial cells and phagocytes, and cathelicidins are secreted from neutrophils in the bloodstream and expressed on epithelial surfaces. The expression of these genes is tightly regulated; they are induced by pathogens and cytokines as part of the host defense response [6, 7], and they can be suppressed by bacterial virulence factors and environmental factors that can lead to increased susceptibility to infection [8, 9]. In contrast, HDP synthesis can be augmented without triggering an extensive inflammatory response by several different classes of small-molecule or dietary compounds as alternatives to antibiotics for disease control and prevention. For example, vitamin D3 potentiates hBD-2 expression via the vitamin D receptor (VDR) pathway [10], and bile salts control the antimicrobial peptide cathelicidin by binding to farnesoid X receptor and nuclear receptors, subsequently up-regulating cathelicidin expression in the human biliary epithelium [11]. The dietary ingredient genistein stimulates CAMP/LL-37 expression through a novel S1P-dependent mechanism, but not the VDR-regulated pathway [12]. In contrast, microbial infection can lead to the induction of AMP cathelicidin and the generation of nitric oxide to kill bacteria via the activation of TLRs and increased MyD88 expression, which activates NF-κB mediated by VDR [13].

Butyrate, a short chain fatty acid (SCFA) that functions as a small molecular inhibitor of histone deacetylase (HDAC), not only functions as an energy source but also plays an immunomodulatory or anti-inflammatory role [14, 15]. The HDP-inducing activity of SCFA is most potent in free fatty acids by inducing cathelicidins and defensins in human, chicken and porcine gastrointestinal or macrophage cells [16, 17]; however, elevated production of HDPs in response to NaB displays species-specific regulation and tissue specificity [17]. The effect of NaB-regulated HDP expression on the kidney has not been reported in any mammalian tissue. PK-15 cells, a porcine kidney cell line that is susceptible to viral or bacterial infection, demonstrate that the innate immune response is responsible for the swift and efficient eradication of pathogens. Moreover, to date, the mechanisms responsible for NaB-induced HDPs have not been fully elucidated. This work highlights the gene expression of HDPs induced by NaB in kidney cells but not the induction of an inflammatory response.

Inhibition of HDAC by trichostatin A (TSA) can enhance the induction of hBD2 expression dramatically without affecting IL-8 expression. This mechanism is supported by the increased phosphorylation of histone H3 on serine S10, preferentially at the hBD2 promoter. This process occurs through activation of the IκB kinase complex, which also leads to NF-κB activation [18]. Our present studies indicate that NaB enhances porcine HDP expression via TLR2 recognition, followed by an IκB α degradation-dependent process and activation of NF-κB p65 phosphorylation independently of MyD88 in kidney cells, in contrast to the increase in IκB α induced by peptidoglycan. However, peptidoglycan can also activate TLR2 and lead to NF-κB p65 phosphorylation and NF-κB activation, but it weakens and strengthens HDP and IL-6 expression, respectively. The goal of this approach is to boost the expression of HDPs but not inflammatory responses, representing a future alternative strategy to antibiotics for the treatment of infections and dysbiosis-driven diseases in humans and animals during periods of an increased incidence of antibiotic resistance.

## RESULTS

### NaB selectively improves AMP gene expression in porcine kidney cells

AMP genes are expressed or regulated in a tissue-specific manner. To evaluate the basal level of the expression of all porcine AMP genes in normal porcine kidney epithelial cells, RT-PCR was performed using specific primers including 29 β-defensin genes and 12 cathelicidin genes, porcine defensins (pBD) including pBD1, pBD2, pBD3, pBD4, pBD107, and pEP2C, among others, and cathelicidins including PR-39, Prophenin-1 or -2 (PF-1 or PF-2), Protegrins-1∼5 (PG-1∼5), and PMAPs (PMAP-23, -36 or -37) (Supplementary Table 1) [5]. The RT-PCR results for all of the above genes revealed those that could be amplified from PK-15 cells in the absence of treatment, including pBD1, pBD2, pBD3, pBD4, pBD108, pBD123, pBD115, pBD128, pBD135, pEP2C, PG-1, and PMAP-23 (Supplementary Figure 1A).

**Figure 1:**
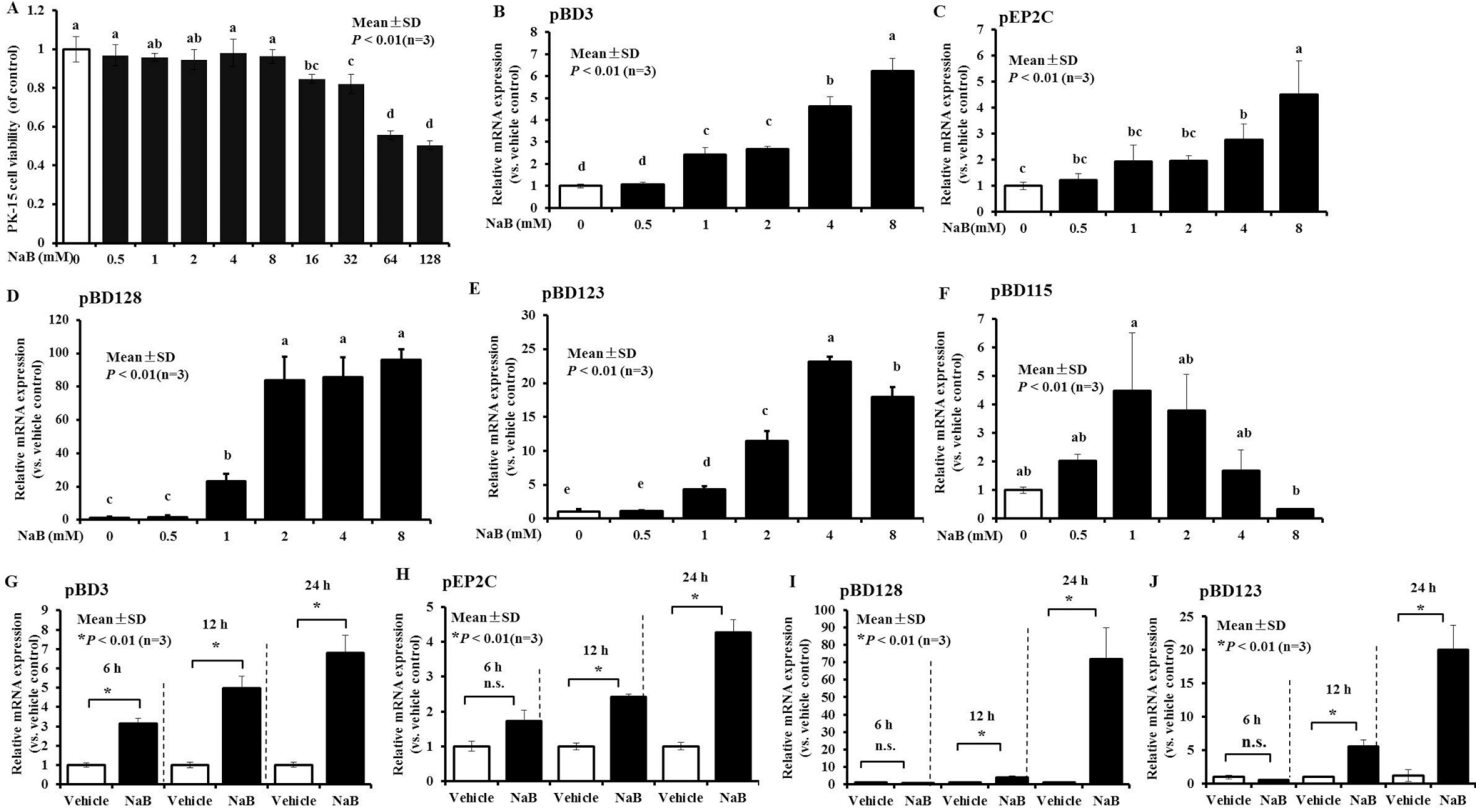
Increased expression of AMP mRNA in porcine kidney cells following NaB (**A**) PK-15 cells were treated with the indicated concentrations of NaB, ranging from 0-128 mM, for 24 hours. Cell viability was measured using CCK-8. All CCK-8 values were normalized to the control serial concentrations of NaB for 24 hours. (**B**–**F**) pBD3, pEP2C, pBD128, pBD123, and pBD115 gene expression were determined by qRT-PCR after treatment with 0, 0.5, 1, 2, 4, and 8 mM NaB for 24 hours. (**G**, **H**) pBD3 and pEP2C gene expression was analyzed by qRT-PCR after incubation with 8 mM NaB for 6, 12, and 24 hours. Similar results were obtained in repeated experiments (more than two) using different cell preparations. Abbreviations: NaB, sodium butyrate. Means with different letters are significantly different at *P* < 0.01 (B–F). *P < 0.01, using the unpaired Student's *t-test* (**G**, **H**, **I**, and **J**).

To analyze the expression of genes that are induced during the innate immune response, including the above genes encoding AMP after treatment with NaB in porcine kidney cells, sub-confluent PK-15 cell monolayers were stimulated with various concentrations of NaB for 24 hours. RNA was extracted after NaB challenge and analyzed by qRT-PCR. Cell viability was not significantly altered by NaB at concentrations ≤ 8 mM, as assessed by the CCK-8 assay (Figure 1A). In cells without NaB, basal expression levels of the studied genes were confirmed by RT-PCR (data not shown). However, the addition of exogenous NaB (0.5–8 mM) for 24 hours was followed by a significant dose-dependent increase in pBD3 and pEP2C by approximately 5–6-fold in porcine kidney cells (Figure 1B and 1C). Additionally, pBD128 transcripts were up-regulated nearly 80-fold (Figure 1D), pBD123 increased approximately 20-fold, peaking at 4 mM (Figure 1E), and pBD115 increased initially and subsequently decreased (Figure 1F). Other genes were not markedly induced after incubation with exogenous NaB, and the expression of some other AMPs, including porcine defensin (pBD104, 106, -108, -113, -116, 117, -118, -121, 122, 124, 125, 127, 133, 134) and cathilincidins (PF1-2, PR-39, PMAP-36, PMAP-37) could not be detected successfully regardless of the presence of exogenous NaB because of the species-specific differences, gene- and cell type-specific differences, or our ability (data not shown).

Furthermore, we examined the time-dependent effects on pBD3, pEP2C, pBD128, and pBD123 expression, which demonstrated a greater magnitude of induction among all genes. Our results revealed a remarkable time-dependent induction of pBD3 following treatment of the cells with 8 mM NaB at 6, 12, and 24 hours (Figure 1G). A clear time-dependent response to NaB was observed for pEP2C expression, in which a marginal up-regulation was observed at 6 hours but a dramatic difference was detected at 12 and 24 hours (Figure 1H). Similarly to pEP2C, pBD128 and pBD123 exhibited a significant increase at 6, 12, and 24 hours in a time-dependent manner (Figure 1I and 1J).

### Inhibition of histone deacetylase plays a distinct role between the increase in NaB-mediated pBD3/pEP2C expression and TSA-mediated pBD1/pBD2 in porcine kidney cells

**Figure 2:**
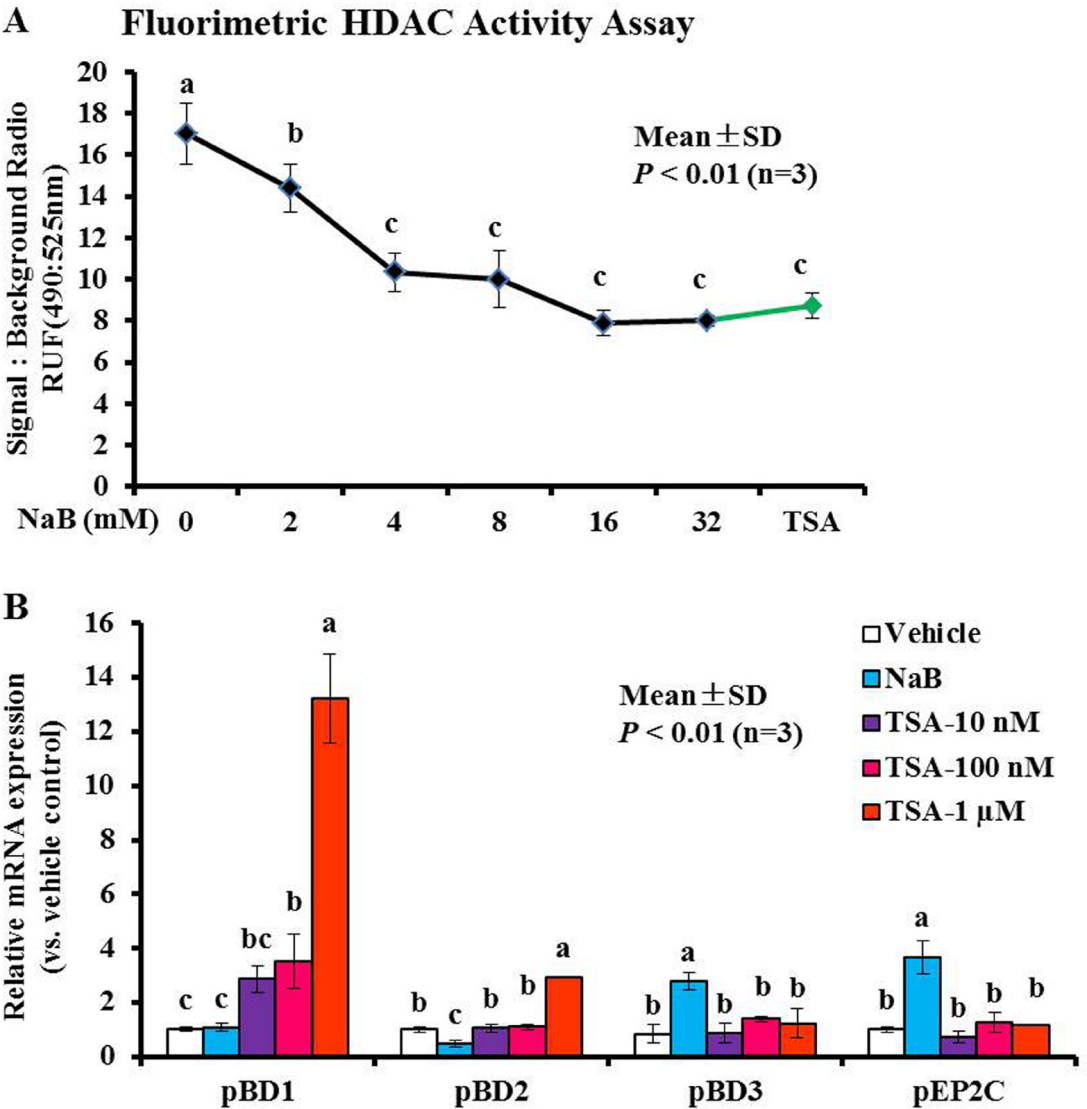
Modulation of histone acetylation activity and AMP gene expression in response to NaB or TSA (**A**) HDAC activity was monitored by excitation at 490 nm and emission at 525 nm. (**B**) PK-15 cells were treated using various concentrations of TSA. Bars represent means with SD of three independent experiments; means with different letters are significantly different at *P* < 0.01.

Butyrate, a four-carbon short-chain fatty acid (SCFA) that is a typical inhibitor of histone deacetylase (HDAC) (termed HDACis), can specifically inhibit class I/II HDAC enzyme activity. Based on the above observations, we sought to evaluate the molecular mechanisms leading to the enhanced induction of pBD3 and pEP2C expression in response to NaB treatment. Therefore, we first ascertained whether NaB attenuated HDAC enzymes activity in PK-15 cells. As expected, the Amplite™ fluorimetric HDAC activity assay revealed a significant dose-dependent inhibition of HDAC enzyme activity following NaB treatment of PK-15 cells (Figure 2A). Concomitantly, a broad-spectrum HDAC inhibitor, trichostatin A (TSA), at 1 μM also showed the anticipated significant inhibition, and compared with vehicle, the total reduction of TSA at 1 μM was similar to that observed with 8 mM NaB. There were no significant differences between NaB at 8 mM and TSA at 1 μM (*P* > 0.01) (Figure 2A). We further determined the changes in AMP gene expression after treatment with 8 mM NaB and serial dilutions of TSA (10 nM, 100 nM, 1 μM) by qRT-PCR. The results showed that treatment of the cells with TSA could only increase the expression of pBD1 and pBD2 but not of pBD3 or pEP2C (Figure 2B). Herein, TSA concentrations ≤ 1 μM did not significantly alter cell viability (Supplementary Figure 2). Taken together, these results showed that the HDAC inhibitors NaB or TSA could elevate AMP gene expression while inhibiting HDAC activity in porcine kidney cells, but the type of AMP induction was different, further indicating that NaB and TSA elevated AMP expression via different mechanisms. Histone deacetylase inhibition played a distinct role in TSA-mediated pBD1/pBD2 expression during the increase in NaB-mediated pBD3/pEP2C in porcine kidney cells.

### p50 in the NF-κB pathway plays an important role, whereas AMP expression is ameliorated by NaB in PK-15 cells

**Figure 3:**
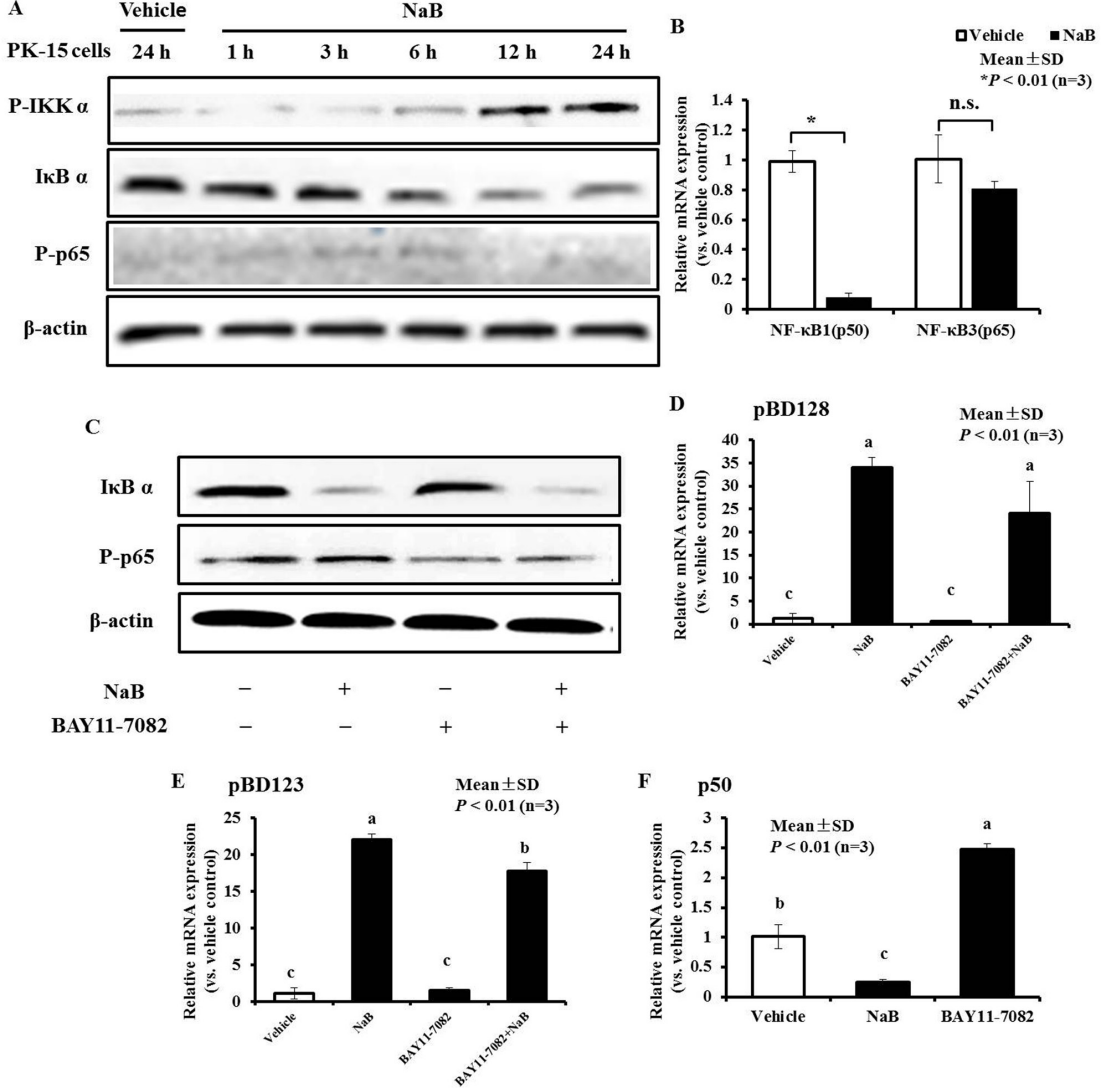
NF-κB pathway plays an important role, while AMP expressionwas ameliorated by NaB in PK-15 cells (**A**) Immunoblot analysis of total IκB α expression and p65 phosphorylation in cells pretreated or not with 8 mM NaB at the indicated time points. Western blots were performed using antibodies directed against IκB α and p65 phosphorylation marks. Vehicle, without NaB treatment cells for 24 h; NaB, cells challenged with NaB for 1, 3, 6, 12, and 24 hours; “P” prefix, phosphorylation. (**B**) qRT-PCR analysis of NF-κB1 (p50) and NF-κB3 (p65) transcription in cells treated with 8 mM NaB. Data were analyzed using the unpaired Student's *t-test* at *P* < 0.01. *P < 0.01; n.s., not significant. C. Immunoblot analysis of total IκB α expression and p65 phosphorylation in cells pretreated or not for 3 h with 2.5 μM BAY11-7082, and then treated with 8 mM NaB for 24 hours. D, E. qRT-PCR analysis of pBD3, pEP2C, pBD128, and pBD123 transcription in cells treated with or without 8 mM NaB and 2.5 μM BAY 11-7082. Bars represent means with SD of three independent experiments. Means with different letters are significantly different at *P* < 0.01. F. qRT-PCR analysis of NF-κB1 (p50) transcription in cells treated with 8 mM NaB or 2.5 μM BAY 11-7082. Bars represent means with SD of three independent experiments. Means with different letters are significantly different at *P* < 0.01.

Next, we investigated the signaling pathway through which NaB mediated HDAC inhibition to induce pBD3 expression. Several studies have demonstrated that canonical histone H3 phosphorylation of HDAC inhibition often occurs through activation of the alternative IKKα/β pathway. IKKα functions as a histone H3 (Ser10) (H3S10) kinase that regulates the structure of chromatin and facilitates gene expression [19, 20]. Furthermore, the phosphorylated IKK complex phosphorylates NF-κB, inhibiting protein IκB α and resulting in its degradation. This process releases NF-κB and allows its translocation into the nucleus [21]. By immunoblotting using an antibody to detect phosphorylation-IKK α and total IκB α, we directly observed dramatic increase of IKK α (phosphor-Ser180) and IκB α degradation in PK-15 cells in response to NaB treatment from 6 to 24 hours, and NF-κB3 (RelA, p65) phosphorylation was occurred (Figure 3A). Simultaneously, the NF-κB1 (p50) and NF-κB3 (RelA, p65) mRNA levels were detected after PK-15 cells were treated with NaB at 8 mM for 24 hours, and the results showed that the mRNA abundance of NF-κB p50 was significantly decreased. In contrast, there were no obvious changes in p65, demonstrating that the NF κB pathway was activated in PK-15 cells by NaB via modification of p65 protein phosphorylation, but not an increased level of transcription (Figure 3B).

The compound BAY 11-7082, an inhibitor of NF-κB, was then employed to inhibit IκB α degradation and p65 phosphorylation induced by NaB, to further explore the relationship between NF-κB pathway activation and the up-regulation of AMP expression. BAY 11-7082 could not inhibit IκB α degradation in PK-15 cells regardless of the presence of NaB (Figure 3C), but it could suppress p65 phosphorylation under either condition. Interestingly, BAY 11-7082 could not suppress pBD3 and pEP2C induction by NaB (data not shown), but there is an inhibiting trend on the expression of AMP pBD128 induced by NaB and but not significantly (Figure 3D). However, blockade of BAY11-7082 could significantly but not completely suppress the expression of AMP pBD128 and pBD123 induced by NaB (Figure 3E). Our results also showed that BAY 11-7082 could enhance NF-κB1 p50 transcription, in contrast to NaB (Figure 3F). Thus, NF-κB pathway activation by NaB might occur through p50 attenuation, which could prevent p65 phosphorylation, and BAY11-7082 might suppress AMP pBD128 and pBD123 expression induced by NaB by reversing p50 reduction by NaB. As a control, cellular viability was not affected by the inhibitor BAY 11-7082 or co-incubation with NaB for 24 hours (data not shown). Collectively, these results indicate that the increase in pBD3 and pEP2C expression is mediated by NaB, potentially through IκB α degradation and p65 subunit phosphorylation followed by NF-κB pathway activation. However, the mechanism underlying the regulation of pBD128 and pBD123 differed between pBD3 and pEP2C. BAY11-7082 reversed the decrease in p50 and p65 phosphorylation leading to NF-κB pathway activation induced by NaB, thus diminishing the expression of pBD128 and pBD123 induced by NaB.

### NaB enhances AMP but not inflammatory cytokine expression

**Figure 4:**
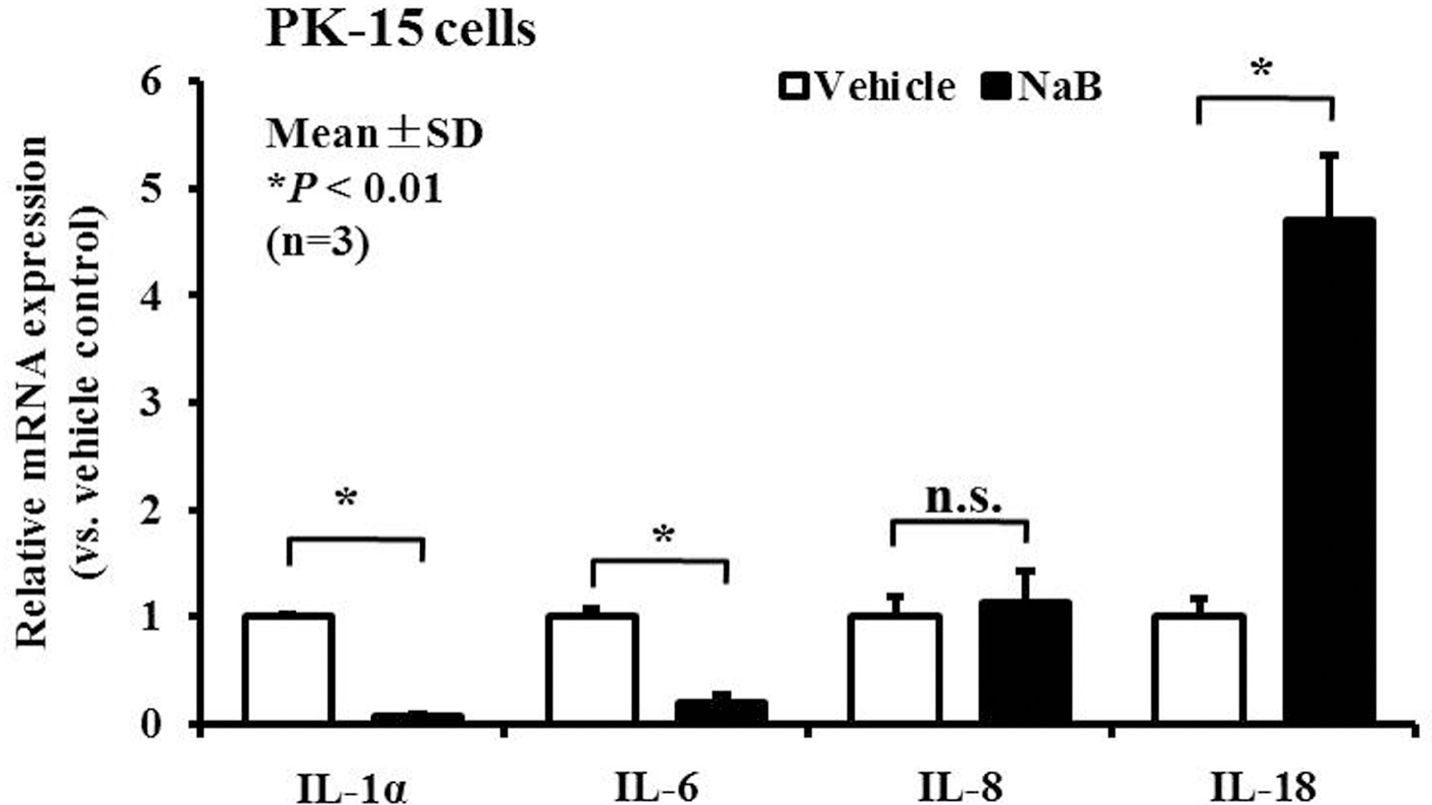
NaB controls inflammatory cytokine expression in PK-15 cells qRT-PCR analysis of inflammatory cytokines, including IL-1α, IL-6, IL-8, and IL-18 mRNA expression, in PK-15 cells treated with 8 mM NaB. *P < 0.01; n.s., not significant at *P* < 0.01.

Some reports suggest that AMP gene expression may be regulated during inflammatory responses in response to invasion by pathogenic microorganisms [22]. Therefore, in addition to molecular features, we examined other innate immune response genes, including genes encoding inflammatory cytokines and chemokines during the enhanced induction of AMP expression following NaB-mediated HDAC inhibition. To achieve this goal, PK-15 cells were treated with NaB for 24 hours, and total RNA from the cytoplasm was harvested. The transcription levels in cells treated with or without 8 mM NaB were measured by qRT-PCR. Interestingly, in addition to AMPs, we identified enhanced expression of IL-18 in cells treated with NaB compared with untreated cells. In contrast, the levels of inflammatory cytokine IL-1α and IL-6 were decreased markedly, but no significant regulation of the levels of the gene encoding the chemokine IL-8 was detected (Figure 4). Taken together, these results suggest that NaB not only up-regulated AMP expression but also had an enormous potential to regulate inflammatory cytokines expression.

### NaB mediates the up-regulation of AMP expression independently of the cell density or serum

**Figure 5:**
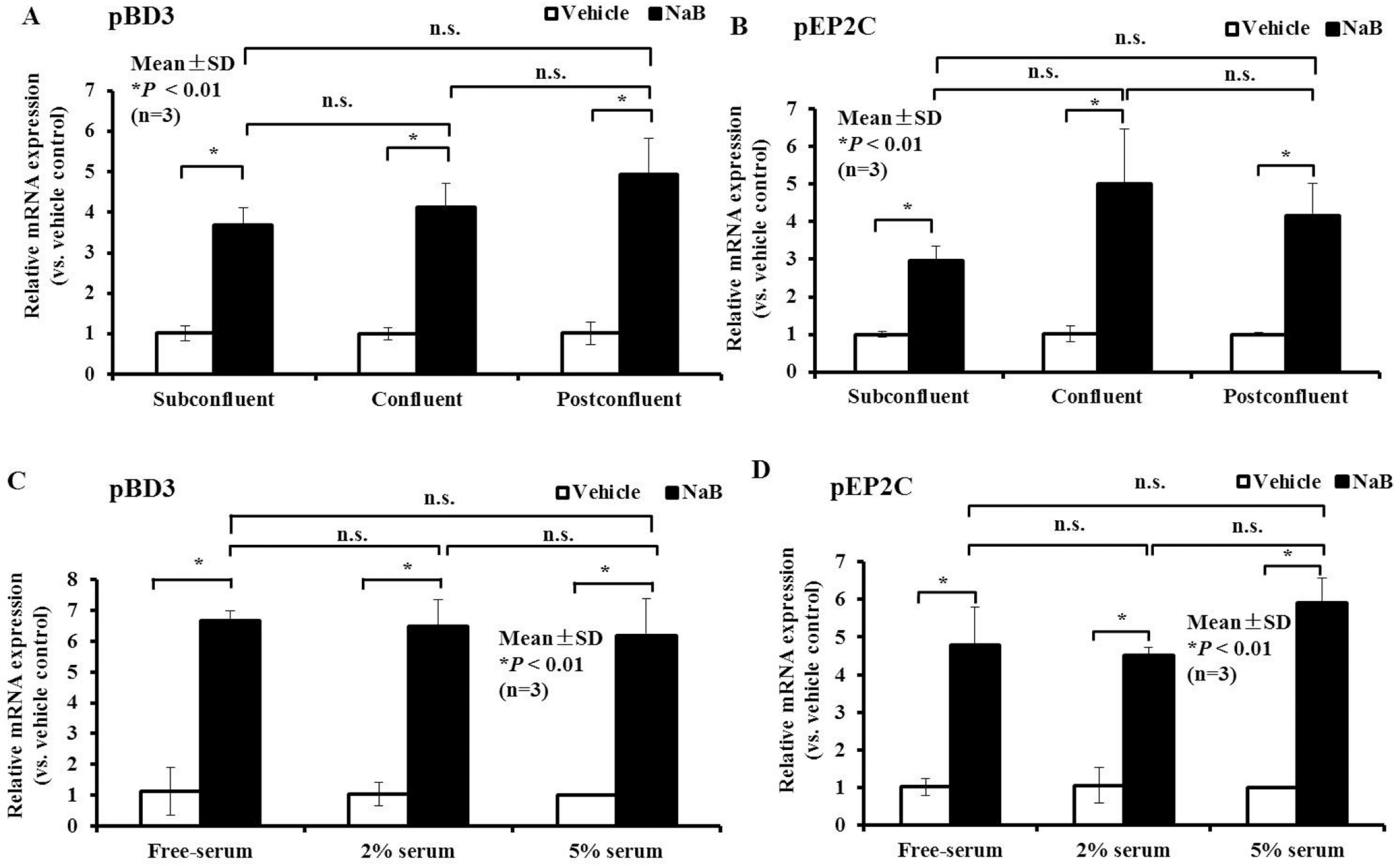
Effect of cell density or serum concentration on NaB governs the up-regulation of AMP expression (**A**, **B**) qRT-PCR analysis of pBD3 and pEP2C transcription at different cell densities treated with 8 mM NaB. (**C**, **D**) qRT-PCR analysis of pBD3 and pEP2C transcription in cells cultured in different serum concentrations and treated with 8 mM NaB. *P < 0.01; n.s., not significant at *P* < 0.01. Data were analyzed using the unpaired Student's *t-test*.

PK-15 cells typically have suprabasal features and can form basal layers with tight junctions. Herein, different densities were used to mimic different degrees of confluence, including sub-confluence, confluence, and post-confluence. Regardless of the degree of confluence, a dramatic increase was observed in pBD3 and pEP2C mRNA expression after NaB treatment. The degree of confluence had no significance effect (*P* < 0.01, Figure 5A and 5B). Moreover, pBD3 and pEP2C mRNA expression were up-regulated in the presence and absence of serum (Figure 5C and 5D). Based on the above results, NaB has a strong capacity to enhance AMP mRNA expression independent of the cell density or serum.

### Role of MAPKs in NaB-mediated AMP up-regulation

**Figure 6:**
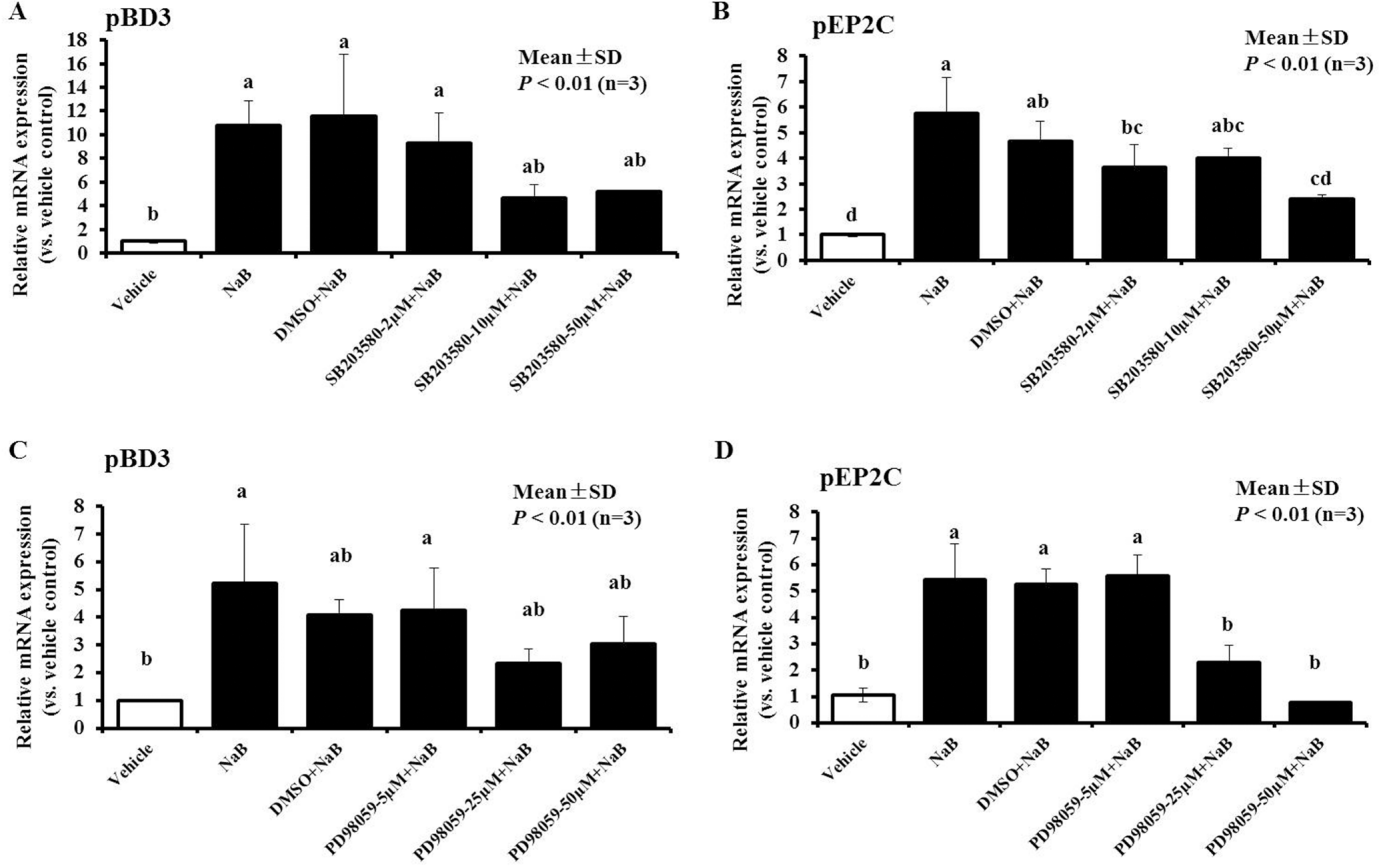
Effect of MAPK inhibitors on the up-regulation of NaB-mediated pBD3 and pEP2C expression PK-15 cells were pretreated with a series of concentrations of the ERK1/2 inhibitor PD98059 or p38 MAPK inhibitor SB203580 and DMSO at a dose no less than the dose required to solve SB 203580 or PD98059 for 5 hours prior to incubation with 8 mM NaB. (**A** and **C**) PD98059; (**B** and **D**) SB203580. Bars represent means with SD of three independent experiments. Means with different letters are significantly different at *P* < 0.01.

We attempted to investigate the mechanism by which NaB leads to induced AMP expression while mediating HDAC inhibition. Some studies have demonstrated that canonical phosphorylation of histone H3 occurs through activation of the MAPK signaling pathway, and activation of Erk1/2, p38, and SAPK/JNK can induce an increase in AMP gene expression [23]. Therefore, PK-15 cells were incubated with NaB (8 mM) in the presence of p38 MAPK or ERK1/2 kinase inhibitor to block the Erk1/2 and p38 intracellular signaling pathways during NaB-induced AMP mRNA expression. PK-15 cells were pre-treated with the p38 MAPK inhibitor SB203580, the ERK1/2 inhibitor PD98059 or DMSO, which is the solvent for SB203580 and PD98059, for 5 hours before incubation with NaB. In this study, we focused on the epigenetic regulation of the gene encoding AMP pBD3 and pEP2C. The greatest effects were obtained using the p38 MAPK inhibitor SB203580 at 10 or 50 μM, which resulted in a 50% decrease in pBD3 expression, and at 50 μM, which also resulted in a 50% decrease in pEP2C expression compared with cells treated with NaB or pre-treatment with DMSO alone for 24 hours (Figure 6A and Figure 6B). However, 25 μM PD98059 significantly decreased the enhanced expression of pBD3 induced by NaB; furthermore, 25 μM and 50 μM PD98059 effectively decreased the up-regulation of pEP2C expression in a clear dose-dependent manner compared with the pre-treatment with NaB alone (Figure 6C and 6D).

### Expression of antimicrobial peptide genes is still improved by NaB in porcine kidney cells upon TLR2 activation

**Figure 7:**
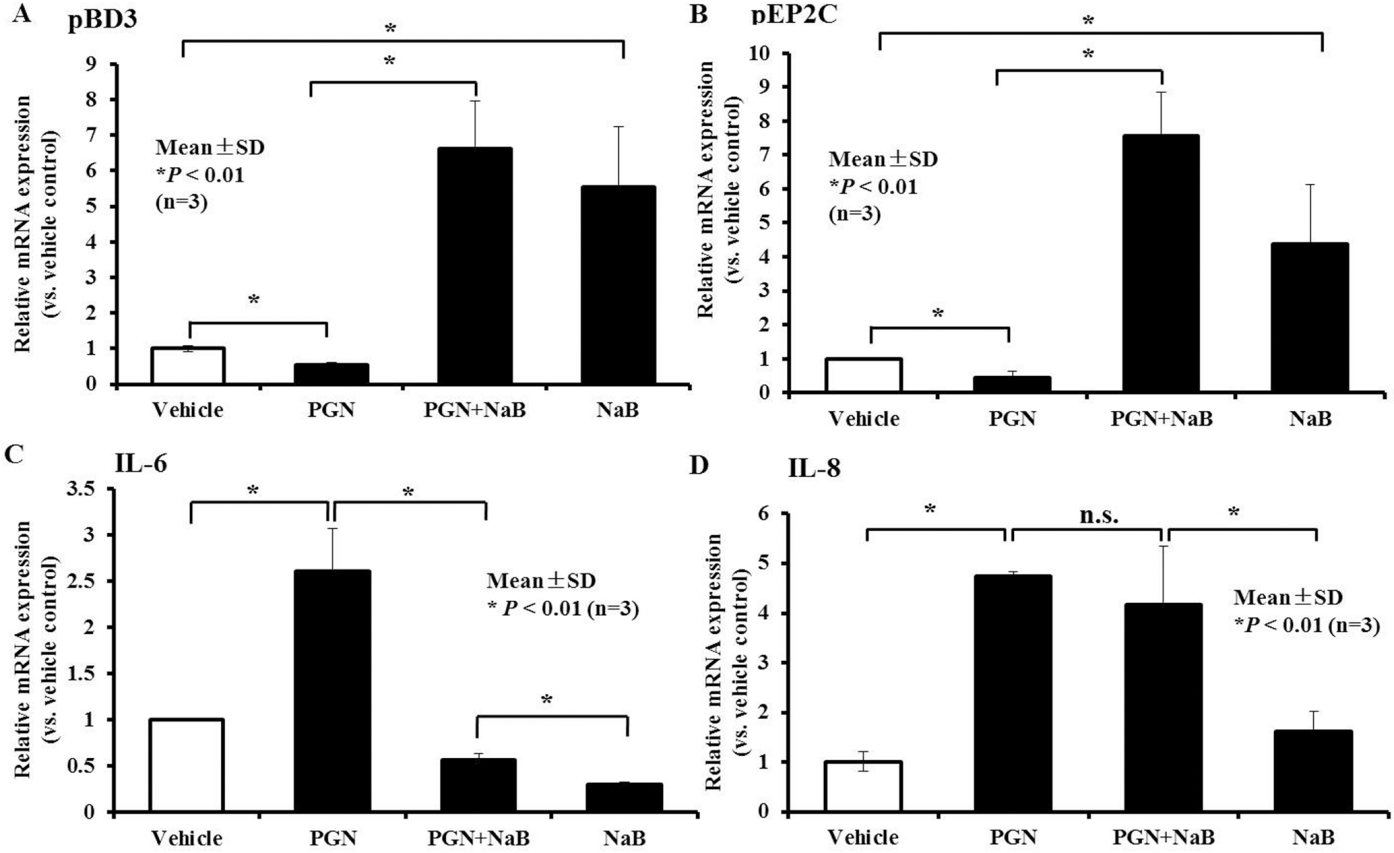
Effect of NaB on porcine kidney cells in the presence of the TLR2 ligand peptidoglycan qRT-PCR to detect changes in gene transcription of (**A**) pBD3, (**B**) pEP2C, (**C**) IL-6, and (**D**) IL-8 in cells treated with 8 mM peptidoglycan and 250 ng/ml peptidoglycan for 24 hours. n.s., not significant at *P* < 0.01; *P < 0.01 (A and B). Data were evaluated using the unpaired Student's *t-test*. “PGN” represent peptidoglycan.

To further assess the up-regulation of AMP expression and the diminished inflammatory response to NaB in porcine kidney cells, PK-15 cells were treated with the TLR2 ligand peptidoglycan to mimic the inflammatory response mediated by TLR2, such as during gram-positive bacterial infection. The expression of TLRs and inflammatory cytokines or AMPs, including TLR2, IL-6, IL-8 and pBD3, and pEP2C were detected after peptidoglycan stimulation for 24 hours in PK-15 cells. As expected, TLR2 expression was significantly increased in peptidoglycan (≥ 50 μg/mL)-treated-PK-15 cells (Supplementary Figure 3A). Although peptidoglycan stimulation for 24 hours could activate TLR2 expression and increased IL-6 and IL-8 inflammatory cytokine levels, the mRNA levels of the AMP genes pBD3 and pEP2C were significantly decreased (Supplementary Figure 3B–3E). Next, we analyzed the expression of AMP and proinflammatory pathways following 8 mM NaB treatment and concomitant stimulation with 250 μg/mL peptidoglycan for 24 hours. RNA was extracted and analyzed by qRT-PCR. Strikingly, NaB not only inhibited the down-regulation of pBD3 and pEP2C induced by peptidoglycan, but it also increased pBD3 or pEP2C expression, ranging from approximately 6–8-fold in comparison to the vehicle without any treatment (Figure 7A and 7B). Moreover, NaB significantly inhibited the increase in IL-6 induced by peptidoglycan to levels even lower than the vehicle (Figure 7C). However, the increase in IL-8 was not influenced by NaB (Figure 7D). Collectively, these data validate that NaB has a strong ability to improve AMP expression during the innate immune response, particularly during inflammatory response inhibition mediated by the TLR2 ligand peptidoglycan.

### Role of TLR2 in AMP expression mediated by peptidoglycan and NaB in PK-15 cells

**Figure 8:**
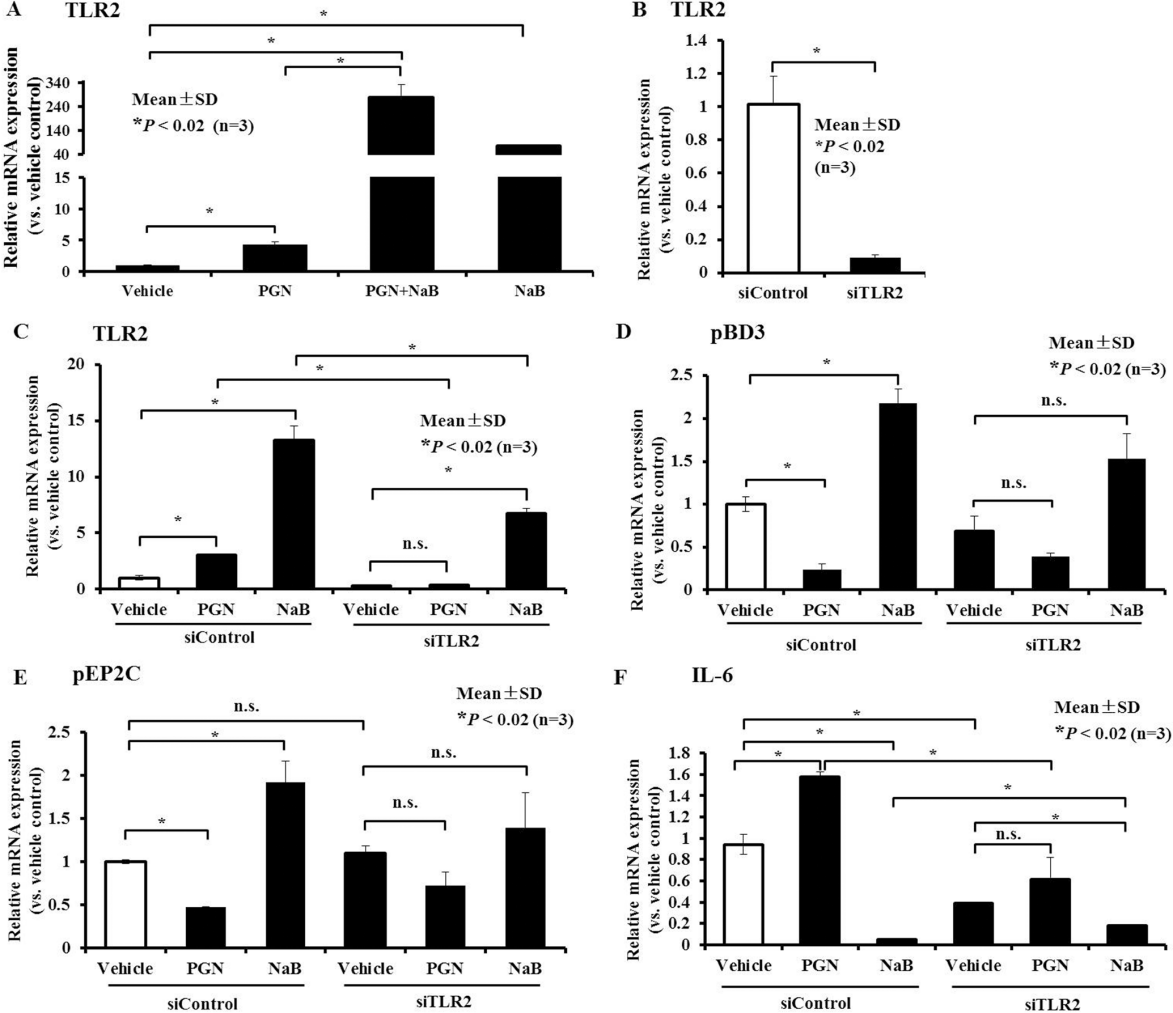
Role of TLR2 in AMP expression control by peptidoglycan and NaB in PK-15 cells (**A**) qRT-PCR analysis of TLR2 expression in cells after treatment with 8 mM NaB or 250 μg/mL peptidoglycan or co-incubation with both for 24 hours. (**B**) PK-15 cells were transfected with siRNA targeting TLR2, and then qRT-PCR was performed to detect TLR2 gene expression. (**C**–**F**) PK-15 cells were transfected with siRNA against TLR2 or siControl, and 6 hours post-transfection, the culture medium was replaced with medium containing 250 μg/mL peptidoglycan or 8 mM NaB for 24 hours. qRT-PCR was then performed to detect TLR2 (C), pBD3 (D), pEP2C (E), and IL-6 (F) transcript levels. Asterisks (*) represent statistical significance at *P* < 0.02, n.s., not significant. “PGN” represent peptidoglycan.

To investigate the upstream mechanism(s) responsible for changes in peptidoglycan-induced AMPs and inflammatory factors mediated by NaB, we first assessed the potential involvement of the TLR2-dependent pathway. The qRT-PCR results showed that TLR2 expression increased following treatment with peptidoglycan (Figure 8A). However, surprisingly, NaB not only did not decrease peptidoglycan-induced TLR2 activation, but it also increased it synergistically together with peptidoglycan (Figure 8A). Simultaneously, NaB also drastically augmented TLR2 expression (Figure 8A). It is interesting that TLR2 could be activated by both peptidoglycan and NaB, but an opposite trend in AMP regulation was observed. This result also validated that changes in TLR2 expression were not the direct cause of the enhanced AMP expression, suggesting that NaB may reverse the down-regulation of AMP expression and alterations of inflammatory factors in response to peptidoglycan via a TLR2-independent pathway.

However, to assess whether the control of AMP expression induced by NaB or peptidoglycan alone was involved in TLR2 activation, we directly assessed whether TLR2 silencing using siRNA could alter NaB and peptidoglycan-mediated regulation of AMPs or inflammatory factors. In this study, TLR2 expression significantly declined in porcine kidney cells transfected with siRNA to specifically knockdown TLR2 (Figure 8B). Basal expression levels of both peptidoglycan and NaB continued to concomitantly enhance TLR2 expression with control siRNA, as expected, and activation of TLR2 expression by peptidoglycan was significantly altered after silencing of the expression of TLR2 receptor. Although NaB still significantly elevated TLR2 expression, compared with cells treated with control siRNA, TLR2 expression declined prominently in porcine kidney cells treated with siRNA against TLR2 following NaB (Figure 8C).

Thereafter, we further examined the control of AMPs by NaB and peptidoglycan after knocking down TLR2, and our results showed that inhibition of pBD3 and pEP2C expression by peptidoglycan was partially restored and pBD3 declined in cells stimulated with 500 μg/mL peptidoglycan (*P* < 0.01) after transfection with siRNA Control (siControl). However, after silencing TLR2, the decrease in pBD3 was not significant, and pEP2C was similar to pBD3 (Figure 8D and 8E). Concomitantly, augmentation of the expression of both NaB-treated pBD3 and pEP2C after knockdown of TLR2 was reduced in response to control siRNA transfection compared with vehicle without NaB, although this difference was not significant (Figure 8D and 8E). In contrast, knockdown of TLR2 expression not only decreased IL-6 production in untreated cells, but it also relieved the increase in IL-6 mRNA induced by peptidoglycan stimulation and decreased the induction in response to NaB addition (Figure 8F). These findings indicated that NaB reversed the changes in AMPs and inflammatory factors in response to peptidoglycan, not through the inhibition TLR2 activation by peptidoglycan but through other targets. However, TLR2 expression plays an important role in regulating expression of the AMPs pBD3 and pEP2C and the inflammatory factor IL-6 in response to peptidoglycan and NaB treatment alone.

### NaB reverses the changes in AMPs and inflammatory responses induced by TLR2 ligands by activating the NF-κB pathway

**Figure 9:**
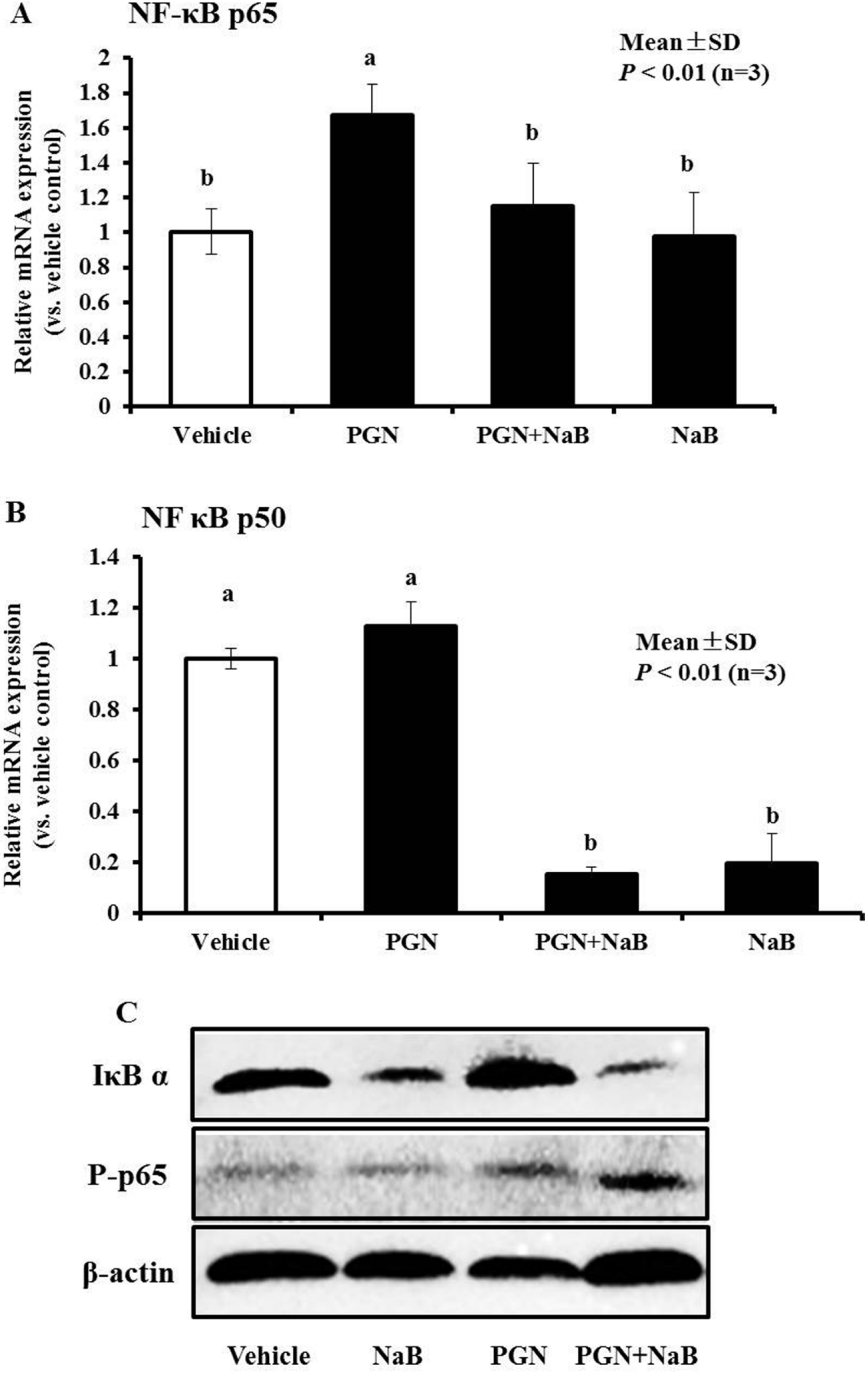
Effect of NaB (8 mM), peptidoglycan (250 μg/mL) or the combination of both on NF-κB signaling in PK-15 cells (**A**, **B**) qRT-PCR was performed to detect p65 and p50 mRNA levels after treatment with peptidoglycan (250 μg/mL), NaB (8 mM), or the combination of both substances for 24 hours. Different letters (a, b, c, and d) indicate significant differences at *P* < 0.01 among the four groups. (**C**) Total proteins were harvested for western blot analysis to detect protein levels of IκB α and p65 phosphorylation in PK-15 cell treated as in (A and B). β-actin served as a loading control. “PGN” represent peptidoglycan.

Exposure of porcine kidney cells to NaB (8 mM) for 24 hours resulted in a reduction of basal p50 and p65 mRNA levels (Figure 3B). Thus, we further explored whether NaB could counteract the regulation of AMPs and IL-6 mediated by peptidoglycan by regulating key NF-κB factors. First, the NF-κB p50 and p65 gene mRNA levels were detected in PK-15 cells after stimulation with peptidoglycan (250 μg/mL), NaB (8 mM), or the combination of both for 24 hours using qRT-PCR. The results showed that NF-κB p65 mRNA was enhanced after peptidoglycan stimulation for 24 hours. However, treatment with NaB (*P* < 0.01) counteracted the peptidoglycan-mediated upregulation of NF-κB p65 mRNA levels, although NaB could not enhance p65 transcription alone (Figure 9A). However, p50 mRNA was not notably changed after peptidoglycan challenge, but NaB could still significantly reduce the p50 mRNA levels (*P* < 0.01, Figure 9B). Western blot analysis revealed the differences in the changes in IκB α expression between the NaB and peptidoglycan treatments. NaB induced IκB α degradation and enhanced p65 phosphorylation; in contrast, peptidoglycan enhanced p65 phosphorylation but also enhanced IκB α expression. Moreover, co-incubation with NaB and peptidoglycan resulted in greater IκB α expression degradation and p65 phosphorylation (Figure 9C). Thus, it was surprising that NaB potentially reversed the effects on AMP and IL-6 expression by inhibiting p65 transcription by peptidoglycan. Additionally, the decrease in p50 may be involved in the improvement in AMPs based on the results obtained for NaB alone and NaB combined with peptidoglycan. In addition, the degree of IκB α degradation and p65 phosphorylation was closely related to the enhancement of AMP expression by NaB.

### Recombinant plasmids bearing the pBD3 gene (mature peptide) can be successfully overexpressed and regulate cytokines in PK-15 cells

**Figure 10:**
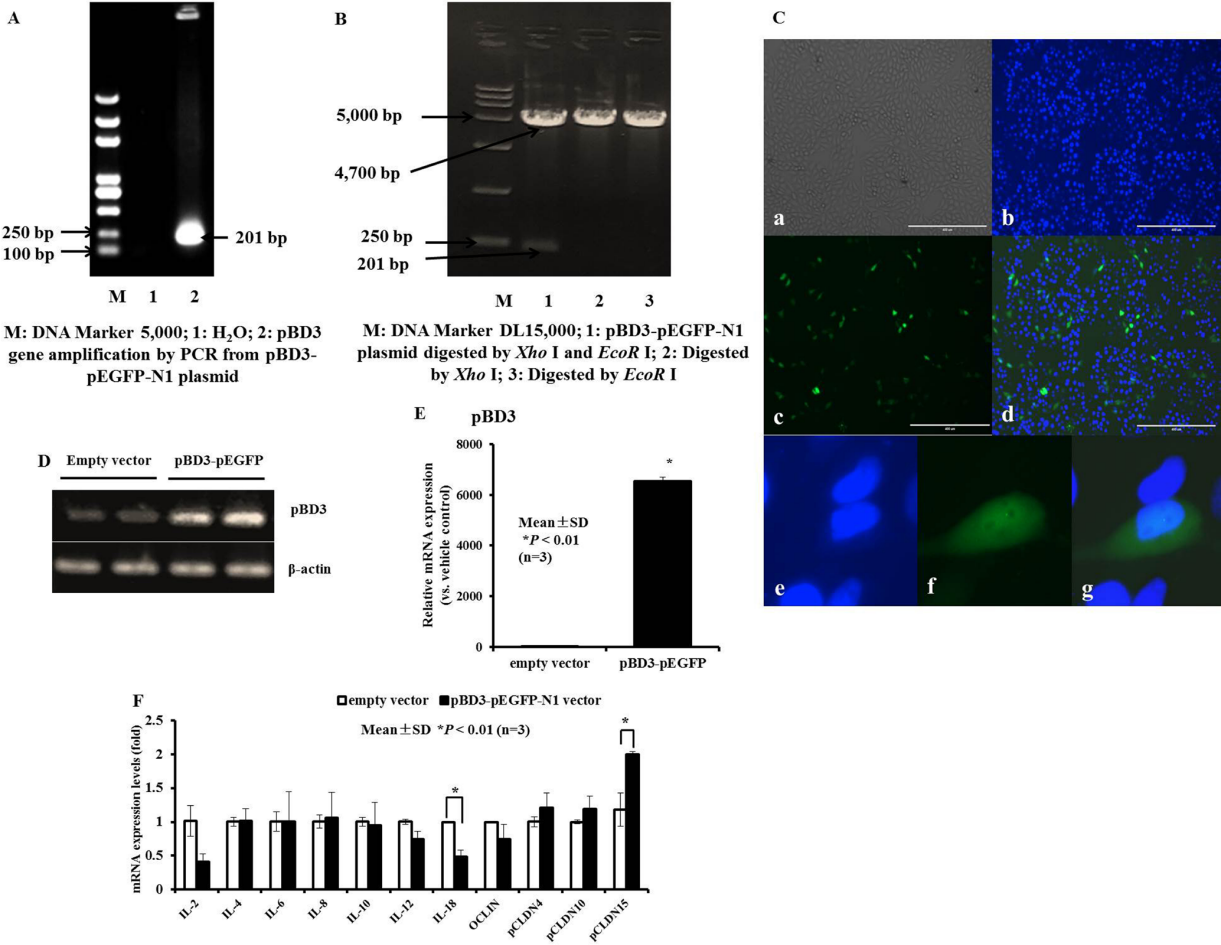
Construction of eukaryotic recombinant expression plasmids pBD3-pEGFP-N1 and expression of pBD3 fusion protein, and the effect of transient overexpression of pBD3 on cytokine, chemokine and tight junction protein production in PK-15 cells (**A**) Restriction enzyme digestion analysis of plasmid pBD3-pEGFP-N1. An inserted fragment of approximately 201 bp was separated by 1% agarose gel electrophoresis following digestion of pBD3-pEGFP-N1 with *Xho* I and *Eco*R I. Lane M, DL15, 000 DNA Marker; Lane 1, pBD3-pEGFP-N1 digested with *Eco*R I and *Xho* I; Lane 2-3, pBD3-pEGFP-N1 digested with *Xho* I and *Eco*R I, respectively. (**B**) pBD3 gene amplification in pBD3-pEGFP-N1 plasmids was analyzed by RT-PCR. Lane M, DL5, 000 DNA Marker; Lane 1, H_2_O; Lane 2, pBD3-pEGFP-N1 plasmids. (**C**) Transient expression of the pBD3 fusion protein in PK-15 cells transfected with pBD3-pEGFP-N1 or pEGFP-N1 and analyzed by fluorescence microscopy. pBD3 protein expression observed by green fluorescence was evaluated in PK-15 cells. (**D** and **E**) pBD3 mRNA expression in PK-15 cells was analyzed by semi-quantitative-PCR or qRT-PCR after transient overexpression of the pBD3 fusion protein. F. qRT-PCR analysis of gene expression profiles for cytokine, chemokine and tight junction protein production in cells overexpressing pBD3 protein. Asterisks (*) indicate statistical significance at *P* < 0.01.

To explore whether the increased expression of endogenous AMP gene leads to changes in cytokines involved in innate immunity in PK-15 cells, a eukaryotic expression vector (pEGFP-N1) carrying the pBD3 gene was constructed. The recombinant plasmid pBD3-pEGFP-N1 was amplified by PCR (Figure 10A), digested with *Eco*R I and *Xho* I (Figure 10B) and sequenced (data not shown). The coding sequence of the recombinant plasmid pBD3-pEGFP-N1 was confirmed to be consistent with the inserted target sequence (detailed data not shown). Next, plasmid pBD3-pEGFP-N1 was transiently transfected into PK-15 cells. Expression of the pBD3-pEGFP-N1 fusion protein in PK-15 cells was analyzed by fluorescence microscopy. As shown in Figure 10C, green fluorescence emitted by expression of the pBD3-pEGFP-N1 fusion protein was observed in the cytoplasm of PK-15 cells. Moreover, pBD3 gene overexpression in PK-15 cells was analyzed by RT-PCR (Figure 10D) and qRT-PCR (Figure 10E). The results showed that the target fragment pBD3 levels were markedly elevated in pBD3-transfected PK-15 cells compared with PK-15 cells transfected with the pEGFP-N1 plasmid empty vector.

Moreover, the expression levels of important inflammatory cytokine interleukins (ILs) or the tight junction protein family of claudins (CLDNs) were determined by qRT-PCR in cells that were transiently transfected with the pBD3 fusion plasmid to induce pBD3 gene overexpression. The results revealed markedly lower levels of IL-18 in pBD3-transfected PK-15 cells than in empty vector-transfected PK-15 cells, a decreasing trend in IL-2, and a marked enhancement of pCLDN15. There were no significant changes in the expression of IL-4, IL-6, IL-8, IL-10, IL-12α, pOCLIN, pCLDN4, or pCLDN10 following the transient transfection of pBD3 genes for 24 hours (Figure 10F). These results demonstrate that pBD3 protein was successfully overexpressed in PK-15 cells after transient transfection for 18–24 hours, resulting in a decrease in IL-18 and an increase in pCLDN15 expression.

## DISCUSSION

To the best of our knowledge, renal epithelial cells maintain a protective barrier function between host innate immunity and invasion by pathogenic microorganisms. Defensins and cathelicidins are two major families of HDPs that are preferentially expressed in mucosal epithelial cells and phagocytic cells. Min-Kyeung Choi et al. reported that HDPs are also broadly expressed in the kidney tissues of 2-week-old piglets. However, temporal changes in gene expression are observed between 2- and 5-month-old pigs, and HDPs are expressed in a tissue-specific manner [5]. Herein, we first explored the basal genome expression levels in porcine kidney cells of 29 porcine β-defensin and 12 cathelicidin genes, including all of the reported porcine HDPs [5]. Among these genes, pBD1, pBD2, pBD3, pBD4, pBD108, pBD115, pBD123, pBD128, pBD135 and PG-1 showed basal and constitutive expression by RT-PCR. However, perhaps because of “extremely low” levels, we failed to detect the basal constitutive expression levels of pBD105, pBD112, pBD114, pBD119, and pBD129, among others, in unstimulated cells.

Although many foreign substances possess HDP-inducing activity, species-specific differences and gene- and cell type-specific differences in HDP regulation clearly occur at least in response to SCFAs (e.g., butyrate, valeric acid) [17, 24]. HDACs are chromatin-modifying enzymes that are known to play pivotal roles as transcriptional suppressors. HDAC is involved in the transcriptional regulation of HDP or LL-37 gene expression in human cell lines [18, 25] or in chickens [26, 27].

However, no information is available regarding histone acetylation and AMP gene expression in porcine kidney cells. Here, we observed an increase in AMPs, including pBD3, pEP2C and pBD123, pBD128, and pBD115, in PK-15 cells treated with NaB, which is a SCFA with prominent HDAC inhibition ability that is exerted in a dose and time-dependent manner. pBD3 and pEP2C are concurrently augmented in porcine intestinal epithelial cell, but the magnitude of induction is greater than that in porcine kidney epithelial cells [17]. To our surprise, pBD128 and pBD123 could be increased more than 80-fold and 20-fold, respectively. Among all the analyzed porcine HDPs, pBD1, pBD4, pBD114, PG-1, and PMAP23, were not induced in PK-15 cells (data not shown).

HDACs often participate in the regulation of transcription factors and signal transduction pathways. NaB, a small specific inhibitor of HDACs, can efficiently suppress HDAC activity [14]. As reported, NaB could suppress the activity of HDACs in porcine kidney cell while increasing AMP gene expression. We speculated that the up-regulation of AMP gene expression might be linked to the inhibition of histone deacetylase. Therefore, trichostatin A, another HDAC inhibitor with an entirely different chemical structure than NaB, was used to assess the regulation of AMPs. Unexpectedly, although we observed that TSA mediated HDAC inhibition in porcine kidney cells, in contrast to NaB, it could not induce pBD3 and pEP2C expression. Interestingly, TSA-mediated HDAC inhibition could effectively enhance pBD1 and pBD2 expression. Different HDAC inhibitors have diverse targets, including class I (HDAC1-3 and 8), class II (4–7 and 9–10), class III (SIRT1-7) and class IV (HDAC11), as shown in Table 1 [27]. The differences in gene type specificities between NaB and TSA may be due to the different targets of HDAC inhibition in porcine kidney cells. HDAC inhibition of HDAC1 with the pan-HDAC inhibitor trichostatin A (TSA) could increase hBD1 gene expression and histone H3 acetylation at the hBD1 promoter [28]. The dietary HDAC inhibitor sulforaphane induces human β-defensin-2 (hBD2) in intestinal epithelial cells [29]. The effect on Ac-α-tubulin also differs between NaB and TSA; TSA, but not NaB, causes a marked increase in Ac-α-tubulin expression [30]. Therefore, pBD3 and pEP2C expression induced by NaB occurred via HDAC inhibition in a manner distinct from that of TSA.

**Table 1:** Specificity of HDACs (NaB and TSA)

HDAC inhibitor	Target HDACs
Butyrate	Class I (HDAC1-3 and HDAC8)
	Class IIa HDACs (HDAC4, 5, 7, and 9)
Trichostatin A	Class I (HDAC1-3 and HDAC8)
	Class IIa HDACs (HDAC4, 5, 7, and 9)
	Class IIb (HDAC6 and 10)
	Class IV (HDAC11)

Histone acetylation and deacetylation modifications play a crucial role in chromatin structure, cellular function and transcriptional regulation of gene expression. The acetylation status of histone proteins is determined by the opposing actions of histone acetyl-transferases (HAT) and histone deacetylases (HDAC). HDACs act as transcriptional repressors due to histone deacetylation and consequently promote chromatin condensation, thereby repressing transcriptional activation [18]. One potential candidate for HDAC inhibition is the IKK pathway, which can mediate this process through the IKK α kinase and also activate the transcription of NF-κB-responsive genes by phosphorylating and targeting its inhibitor IκB α for proteasomal degradation [31, 32]. p65 (NF-κB3) and p50 (NF-κB1) are two key subunits of the NF-κB pathway. The p50 subunit lacks a C-terminal transactivation domain (TD), in contrast to p65 with a characteristic transcriptional activation domain. The NF-κB p50-p50 homodimer has been reported to function as a transcriptional repressor [33]. Our results showed a dramatic decrease in p50, unlike p65, which seemed to suggest that NF-κB was activated after NaB treatment. The increase in p65 phosphorylation further suggested that NF-κB was activated. Moreover, an apparent increase in IKK-α phosphorylation and decrease in IκB α protein levels were also observed in kidney cells. The IKK-α phosphorylation and IκB α degradation further support our hypothesis that histone acetylation activated downstream of the NF-κB signal may occur via the phosphorylation of H3S10, which is a predisposing mark for histone acetylation, a marker of active transcription [34]. The NF-κB inhibitor BAY11-7082 does not inhibit the canonical IKK complex or IKK-related kinases, but it suppresses LPS or IL-1-stimulated phosphorylation of the activation loop of IKK β and thereby the degradation of IκB α in a MyD88 (myeloid differentiation factor 88)–dependent signaling network by targeting the ubiquitin system [35]. Herein, we found not only that BAY11-7082 could not degrade IκB α expression in porcine kidney cells in the absence of stimulation but also that it could not prevent the degradation of IκB α induced by NaB. There was no increase but instead a dramatic decrease in MyD88, an adaptor protein during suppression that leads to the degradation of IκB α (data not shown). However, BAY 11-7082 clearly inhibited p65 phosphorylation in cells regardless of the presence of NaB. Interestingly, BAY 11-7082 could not inhibit the up-regulation of pBD3 and pEP2C induced by NaB, but it could inhibit the enhancement of pBD123 and pBD128 expression through p65 phosphorylation. These findings suggest that NF-κB signaling network activation may be a vital part of the mechanism underlying the induction of porcine AMP expression signaling pathways. Additionally, compared with pBD123 and pBD128, pBD3 and pEP2C did not have an equivalent regulatory mechanism. Based on these results, it is worth noting that NaB activation of the NF-κB signaling network differed from that of lipopolysaccharide (LPS) or some inflammatory cytokines. As reported previously, BAY 11-7082 could not inhibit the canonical IKK complex or IKK-related kinases *in vitro*, but it could inhibit LPS-induced IκB α degradation [35]. This result suggested that NF-κB signaling induced by NaB differed from that caused by LPS. Furthermore, the changes in p50 between NaB and BAY11-7082 further demonstrated that lower p50 levels were beneficial for AMP expression enhancement in porcine kidney cells.

Some bacteria and viruses commonly infect host cells by activating the NF-κB signaling network, thereby enhancing the production of downstream proinflammatory cytokines such as IL-1, IL-6, and TNF-α, among others [36]. However, surprisingly, although NaB activated NF-κB signaling, it decreased the production of IL-1 and IL-6 in porcine kidney cells. However, the HDAC inhibitors NaB, TSA, and valproate regulated the expression of IL-18, a cytokine with antitumor and inflammatory regulatory properties in human acute myeloid leukemia cell lines [37]. A recent report also demonstrated that butyrate could mediate the induction of IL-18 in colonic epithelium using GPR109A as a receptor (encoded by *Niacr1*) and that butyrate-GPR109a signaling imparts an anti-inflammatory phenotype to DCs and macrophages [38]. In our study, IL-18 in porcine kidney cells was increased 5-fold compared with the non-stimulated group. IL-18 has been reported to increase the expression of certain AMPs, and treatment with recombinant IL-18 significantly increased mRNA expression of CRAMP in mice [39]. Interestingly, in the following analyses, we found that pBD3 overexpression attenuated IL-18 expression. HDPs were also reported as immune regulators to attenuate pro-inflammatory cytokine production by microbial products. A dual directional regulatory mechanism may be present between AMPs and IL-18 in porcine kidney cells.

When differentiation influences signal transduction, subconfluent cells utilize NF-κB and post-confluent cells utilize MEK1/2, and p38 [40]. In a previous study, the p38 MAPK signaling pathway was found to be involved in butyrate-CAMP gene expression [24]. Our results indicate that the p38 MAPK inhibitor SB203580 and the ERK1/2 inhibitor PD98059 have a small effect on blocking the induction of pBD3 and pEP2C in subconfluent cells in a similar manner.

The ability of AMP to target a broad group of, mostly, pathogens has remained puzzling until recently [41]. Peptidoglycan, an important constituent of the gram-positive bacterial cell wall, is a TLR2 ligand [42] and thus plays a vital role in the activation and regulation of host immune responses [43–45]. To further explore the roles of NaB-induced AMP, we inhibited the inflammatory response in the presence of bacteria. Peptidoglycan was used to stimulate porcine kidney cells to generate a mini immune response model induced by gram-positive bacterial infection. Extracellular peptidoglycan was recognized by the cells via activation of TLR2 expression, which led to the activation of downstream signaling pathway members in the human embryonic kidney HEK293 cell line [42]. In porcine kidney cells, IL-6 mRNA expression spiked after TLR2 activation in response to peptidoglycan exposure for 24 hours. Although the production of inflammatory cytokines is important for mediating the initial host defense response to invading pathogens, an excessive inflammatory response can be detrimental to the host. Thus, TLR-mediated inflammation is a double-edged sword that must be precisely regulated. Herein, NaB could effectively regulate the excessive production of IL-6, thereby maintaining homeostasis. AMP gene expression was abrogated by *Helicobacter pylori* virulence effectors during prolonged infection via the inactivation of EGFR signaling, to evade a key innate mucosal defense pathway and thus support the establishment of persistent infection [8]. Our results showed that mRNA expression of the AMP genes pBD3 and pEP2C was markedly suppressed after peptidoglycan stimulation for 24 hours, which may represent a mechanism employed by Gram-positive bacteria against the host innate immune system. However, it is encouraging that AMP inhibition and IL-6 elevation in response to peptidoglycan were restored in the presence of both peptidoglycan and NaB. Therefore, we found that NaB had a greater ability to up-regulate AMP expression and inhibit IL-6 production, regardless of whether the cells were grown under common conditions or stimulated with peptidoglycan.

TLR2 is a main receptor for microbial products from Gram-positive bacteria, such as peptidoglycan and lipoteichoic acid (LTA) [46]. Exogenous peptidoglycan was recognized by TLR2 in porcine kidney cells. TLR2-silenced PK-15 cells exhibited diminished pBD3, pEP2C down-regulation and IL-6 up-regulation following peptidoglycan stimulation. Therefore, we demonstrated that TLR2 activation played a crucial role in peptidoglycan-stimulated PK-15 cell responses, and it directly regulated the expression of AMPs. We speculated that NaB could inhibit AMP expression by diminishing TLR2 activation induced by peptidoglycan. However, our results confirmed that not only was NaB unable to diminish TLR2 activation but also, in contrast, TLR2 was activated by NaB itself. In a previous report, the membrane abundance and mRNA expression of TLR2 were increased 1.6-fold and 1.7-fold, respectively, by 0.5 mM NaB in bovine mammary epithelial cells [47]. Herein, TLR2 mRNA was induced by NaB in porcine kidney cells by approximately 60-fold, exceeding the fold activation in bovine mammary epithelial cells. TLR2 plays a crucial role in the regulation of AMPs in response to treatment with NaB and peptidoglycan alone by silencing the targeted TLR2. The regulation of AMPs was significantly altered by reduction of the peptidoglycan-induced increase in p65 and the decrease in p50 mRNA, and this regulation played a role in the retro-regulation of IκB α degradation, in contrast to peptidoglycan, thus promoting IκB α degradation. These results also demonstrated that both NaB and peptidoglycan could activate NF-κB signaling, but in distinct ways. Together, these results demonstrate that AMP regulation induced by NaB is a complex process that is regulated in multiple ways.

In our studies, we addressed whether the increase in endogenous pBD3 expression could regulate the expression of other immune regulators. The important inflammatory cytokines interleukins and typical tight junction protein CLDNs were evaluated. Remarkably, a marked decrease in IL-18 and an enhancement of pCLDN15, a classic CLDN, were observed after transient transfection of the pBD3 fusion protein for 24 hours. In a previous report, β-defensin 131 produced though the overexpression of β-defensin 131 plasmid mediated immunoregulation, especially the release of proinflammatory cytokines and chemokines [48] Additionally, overexpression of hBD2 and pBD2 could attenuate inflammation [49, 50]. Although the species and cytokine levels in previous studies were reduced by β-defensin 131 overexpression by 100,000-fold using G418, in the present analysis, pBD3 was overexpressed by only 7,000-fold due to the transient expression in a very short time period. Therefore, the increase in endogenous pBD3 expression could regulate cytokine transcription, supporting a strong immunoregulatory ability.

## MATERIALS AND METHODS

### Reagents, antibodies and cell culture

The pharmacological inhibitor sodium butyrate (NaB) (B5887, Sigma, St. Louis, MO), HDAC inhibitor trichostatin A (TSA) (T1952, Sigma), NF-κB inhibitor BAY11-7082, p38MAPK inhibitor SB203580, and ERK1/2 inhibitor PD98,059 were all purchased from Beyotime (S1523, S1863, S1805, Shanghai, China). Antibodies against phosphor-NF-κB p65 (Ser536) (93H1) rabbit mAb were purchased from Cell Signaling Technology (#3033, USA), and anti-IκB α (SC-371, Santa Cruz), anti-β-actin (13E5) and secondary horseradish peroxidase (HRP)-conjugated anti-rabbit IgG were purchased from Cell Signaling Technology, Inc. (4970, 7077, USA). Peptidoglycan - TLR2 ligand was purchased from In*vivo*Gen (tlrl-pgnb3, USA). The AmpliteTM Fluorimetric HDAC Activity Assay Kit was purchased from AAT Bioquest (13601, Sunnyvale, CA, USA). Plasmids were extracted with the EndoFree Mini Plasmid Kit from TIANGEN BIOTECH (DP118, Beijing, China).

The immortalized porcine kidney epithelial cell line PK-15 was purchased from ATCC (http://www.atcc.org/Products/All/CCL-33.aspx), cultivated in high glucose Dulbecco's Modified Eagle's Medium (DMEM, Gibco, Carlsbad, CA) supplemented with 10% heat-inactivated fetal bovine serum (FBS, 1552680, Bioind) and 1% penicillin/streptomycin (100 U/mL and 100 mg/mL, respectively) (V900929, Sigma, St. Louis, MO) and maintained in a humidified atmosphere at 37°C with 5% CO_2_. The cells were routinely passaged at 80–90% confluence.

### Reverse transcription-polymerase chain reaction (RT-PCR)

Total RNA was isolated from PK-15 cells using TRIzol reagent (94604, Ambion, Life technologies, Carlsbad, USA) according to the manufacturer's instructions, and 1 μg total sample RNA was reverse-transcribed into complementary DNA (cDNA) using M-MLV reverse transcriptase (RR037A, Takara Bio Inc., Shiga, Japan). The PCR primer sequences for the genes analyzed in this study are listed in Supplementary Table 1, and the products were electrophoresed on a 1% agarose gel.

### Cell viability assays

The toxic effects of NaB on PK-15 cells were determined using the Cell Counting Kit-8 (CCK-8) assay (BS350B, Biosharp, Anhui, China). Briefly, approximately 5 × 10^3^ cells were plated in 96-well plates (Corning, USA). After growing the cells to 60% to 70% confluence, they were incubated with different concentrations of NaB at 37°C for 24 hours. Then, 2-(2-methoxy-4-nitrophenyl)-3 -(4-nitrophenyl)-5-(2,4-disulfophenyl)-2H-tetrazolium, monosodium salt (WST-8) reagent was added to the cells according to the manufacturer's protocol, and the absorbance was recorded at 450 nm using an Infinite M200 microplate reader (Tecan, Durham, USA). Mock-treated cells served as a control. Each experiment was performed in triplicate.

### Quantitative real-time PCR

Quantitative real-time PCR (qRT-PCR) was performed to evaluate and quantify the mRNA levels of cytokines in PK-15 cells following the treatment described above for the indicated periods. Briefly, the cDNA samples were analyzed by qRT-PCR using SYBR green I as the fluorescent dye (RR420A, Takara Bio Inc., Shiga, Japan). Relative quantifications of the mRNA expression of the target genes were calculated using the comparative threshold cycle number for each sample (2^−ΔΔCT^). Gene expression was normalized to the corresponding β-actin level. The main primers used for qRT-PCR in this study are shown in Supplementary Table 1 [51–54].

### Histone deacetylase (HDAC) activity assay

The Amplite™ Fluorimetric HDAC Activity Assay Kit was used to measure HDAC activity in PK-15 cells according to the manufacturer's instructions. Briefly, PK-15 cells were plated at a density of 1 × 10^5^ cells per well in 12-well plates and incubated with different concentrations of NaB or TSA, a common HDAC inhibitor serving as a positive control for 24 hours at 37°C. The “0” represents cells without NaB or TSA treatment. Each group was assessed in triplicate. Cell pellets harvested after 24 hours were homogenized in ice-cold RIPA lysis buffer (P0013B, Beyotime, China) containing the complete protease inhibitor phenylmethyl sulfonylfluoride (PMSF) (ST506, Beyotime, Shanghai, China). Protein concentrations were measured using the trace nucleic acid protein analyzer (Implen, Germany). Cell lysates were diluted to an appropriate range containing equivalent amounts of proteins in assay buffer, followed by the addition of 40 μL of diluted cell lysates. Then, 50 μL of HDAC Green™ Substrate working solution was added to each well, and the plate was incubated at room temperature for 45 minutes. The fluorescence intensity at Ex/Em = 490/525 nm was monitored. The fluorescence detected in the blank wells (with assay buffer only) was used as the background fluorescence and subtracted from the values determined for the wells subjected to HDAC Green™ reactions. All fluorescence readings are expressed in relative fluorescence units (RFU), and each experiment was performed in triplicate.

### Western blotting analysis

Cell pellets harvested at the indicated times and from the indicated treatment modes were homogenized in ice-cold RIPA buffer containing complete protease inhibitors (PMSF). Total protein was resolved on a 12% SDS-PAGE gel to detect IκB α protein or β-actin and on an 8% gel to detect phosphorylated p65 protein and then transferred onto polyvinylidene fluoride (PVDF) membranes (Millipore, USA). After blocking with 5% milk in Tris-buffered saline (10 mM Tris-Cl at pH 7.5 and 150 mM NaCl) containing 0.05% Tween 20 (TBST) at 37°C for 2 hours, the membranes were incubated with primary antibody against targeted protein at 4°C overnight, followed by incubation with the corresponding HRP-linked secondary antibodies (dilution, 1:2500) for 1 hour at room temperature. The protein bands were developed using a super enhanced chemiluminescence (ECL) Plus detection system (P1010, Applygen, Beijing, China) and visualized with X-ray film (Clinx Science Instruments Co., Ltd., Shanghai, China). The expression of each protein was normalized to that of β-actin.

### RNA interference for TLR2 gene knockdown

For TLR2 gene knockdown, PK-15 cells were transfected with 160 nM of specific silencing (si) RNA oligonucleotides targeting porcine TLR2 or scrambled oligonucleotides with FAM synthesized by Sangon Biotechnology of China using Lipofectamine 2000 reagent (11668, Invitrogen, Carlsbad, CA, USA) according to the manufacturer's instructions. The scrambled oligo, which had no specific target in the mammalian genome, was used as a control. Briefly, 160 nM siRNA and 1.5 μL of Lipofectamine RNAi max transfection reagent were diluted in separate tubes to 50 μL in serum-free opti-MEM (Gibco) and incubated for 5 min at room temperature. The two solutions were mixed and incubated further at room temperature for 20 min to form siRNA-Lipofectamine complexes (100 μL), and they were transfected in a final volume of 500 μL into 70–80% sub-confluent PK-15 cells for 24 hours before gene knockdown analysis. The knockdown efficiency was analyzed by qRT-PCR. The sequences of specific siRNA oligonucleotides were as follows. TLR-sense: CCAGAUCUUUGAGCUCCAUTT; antisense: AU GGAGCUCAAAGAUCUG GTT; negative control-sense: UUCUCCGAACGUGUCACGUTT; antisense: ACGUG ACACGUUCGGAGAATT [55].

### Construction of eukaryotic expression recombinant plasmids carrying the pBD3 gene

The gene encoding the mature pBD3 gene of *Sus scrofa* (GenBank, NM_214444.1) with the sequence ATGAGGATCCACTACCTTCTCTTTGCCT TGCTCTTCTTGTTCCTGATGCCTCTTCCAGGTAAT GGAAGAATCATAAATA CATTACAAAGGTATTATT GCAAAATAAGACGCGGCCGCTGTGCTGTGCTTGG CTGCCTTCCAAAAGAGGAACAGATAGGTAGCTGT TCTGTGAGTGGCCGAAAATGCTGCCGAAAGAGG AAA, was synthesized as a full-length oligonucleotide without the terminator codon (TGA) using standard solid-phase methods at BGI Biotechnology Company of China. The gene fragment was subcloned into *E. coli* pUC57-simple cloning vector after digestion with *Xho* I and *Eco*R I. Next, the fragment was subcloned into the eukaryotic expression cloning vector pEGFP-N1. After the identification of positive colonies, pBD3-pEGFP-N1 plasmid cDNA was extracted using PCR and mono or dual restriction endonucleases, agarose gel electrophoresis and DNA sequencing. The sequences of the primers used to amplify the full-length pBD3 were forward: 5′-atgaggatccactaccttctctttg and reverse: 5′-tttcctctttcggcagca.

### pBD3 recombinant plasmid transfection and overexpression

PK-15 cells were grown to approximately 80% confluence in 24-well plates and then transiently transfected with the confirmed recombinant plasmid pBD3-pEGFP-N1 at a dose of 0.5 μg/well using Lipofectamine 2000 according to the manufacturer's instructions. Simultaneously, empty-vector pEGFP-N1 plasmid and no transfection were used as negative controls. Twenty-four hours after transfection, the cells were rinsed twice with cold PBS, fixed in 4% formaldehyde in PBS for 30 min at room temperature and quenched with 0.1 M glycine in PBS. The cells were then washed three times with PBS, followed by incubation with DAPI (1:10 dilution in PBS containing 0.1% BSA) for 20 min at room temperature. After washing with PBS, the fluorescence signals were analyzed using fluorescence microscopy. Moreover, the effects of the expression of pBD3 and other immunomodulatory factors following pBD3 protein overexpression were determined by qRT-PCR. The sequences of the primers used are shown in Supplementary Table 2.

### Statistical analysis

All data were obtained from at least three independent experiments and expressed as the means ± standard deviations (SD). The differences and statistical significance of the gene expression ratios of drug-treated versus normal samples were analyzed using an unpaired Student's *t-test* or GLM (General Linear Model of Statistical Analysis System, SAS 9.4.2, 2000). A *P-value* less than 0.01–0.02 was considered statistically significant (*P < 0.01 or 0.02; n.s., not significantly different). Different letters indicate significant difference at *P* < 0.01.

## CONCLUSIONS

**Figure 11:**
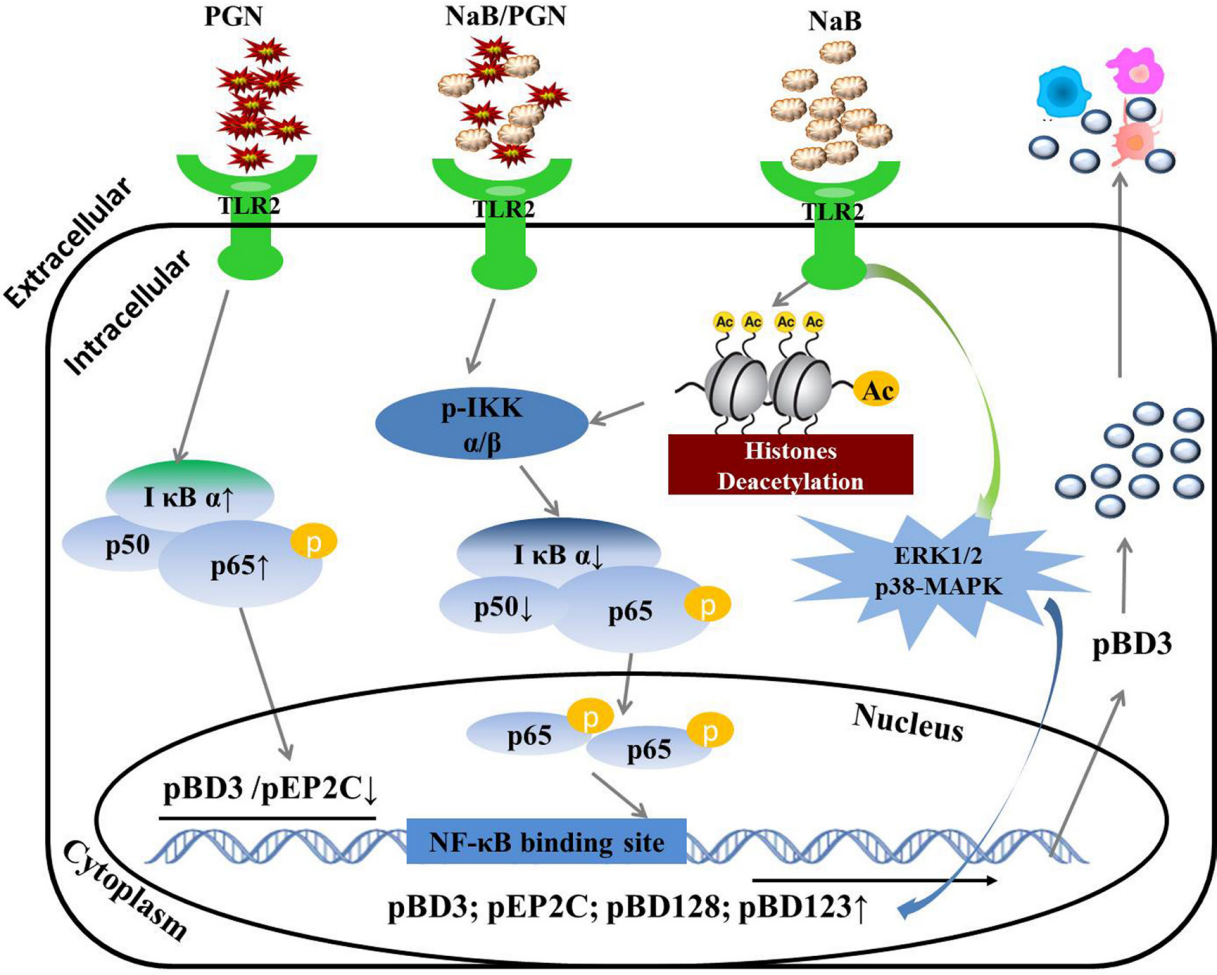
Regulatory mechanism of AMP gene expression induced by NaB in porcine kidney cells NaB inhibited HDAC activity and improved AMP expression, even following challenge with the TLR2 ligand peptidoglycan. The mechanism: NaB leads to IκB α degradation, a reduction of p50 transcription and an increase in p65 phosphorylation, subsequently activating the NF-κB pathway and leading to enhanced AMP transcription via TLR2. The MAPK pathway also plays a role in the regulation of AMPs by NaB. NaB reversed the down-regulation of AMPs induced by the TLR2 ligand peptidoglycan by inhibiting the levels of p65 and decreasing p50 transcription and even IκB α degradation, which are key factors in the NF-κB pathway. Finally, overexpression of pBD3 protein regulated the expression of other cytokines in porcine kidney cells.

To the best of our knowledge, this study is the first to assess the regulation of AMPs by NaB in porcine kidney cells. We demonstrated that NaB improved AMP expression while inhibiting HDAC activity, even following challenge with the TLR2 ligand peptidoglycan, at different degrees of confluence and with reduced serum concentrations. We also further showed the underlying mechanism: NaB leads to IκB α degradation, a reduction of p50 transcription and an increase in p65 phosphorylation, subsequently activating the NF-κB pathway and enhancing AMP transcription via TLR2. Moreover, the MAPK pathway also plays a role in AMP regulation by NaB. Furthermore, NaB reverses the down-regulation of AMPs induced by the TLR2 ligand peptidoglycan by inhibiting p65 levels and decreasing p50 transcription and even IκB α degradation. Finally, we overexpressed pBD3 protein in porcine kidney cells and showed that it could regulate the expression of other cytokines, as shown in Figure 11. Thus, our results provide a novel approach demonstrating that NaB increases the expression of AMPs but not the inflammatory response in porcine kidney, suggesting its potential as a future antibiotic alternative strategy to prevent and treat infections during a period of increasing antibiotic resistance.

## SUPPLEMENTARY MATERIALS FIGURES AND TABLE


