# Supplementary material for: Sodium butyrate improves porcine host defense peptide expression and relieves the inflammatory response upon toll-like receptor 2 activation and histone deacetylase inhibition in porcine kidney cells
Source: Oncotarget. 2017 Feb 24;8(16):26532–51. doi: 10.18632/oncotarget.15714 (PMC5432277; doi:10.18632/oncotarget.15714)
Supplement: Supplementary file 1 [file oncotarget-08-26532-s001.pdf]

Sodium butyrate improves porcine host defense peptide expression and relieves the inflammatory response upon toll-like receptor 2 activation and histone deacetylase inhibition in porcine kidney cells

Supplementary Materials

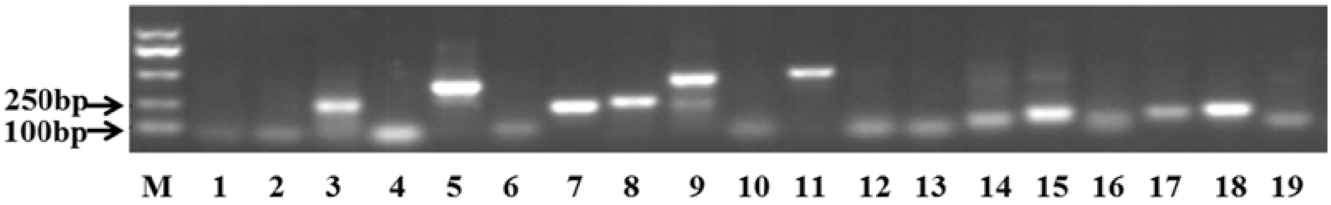

Supplementary Figure 1: HDP gene amplification in PK-15 cells was analyzed by RT-PCR. Lane M, DL5, 000 DNA Marker; Lane 1: pBD105; Lane 2: pBD112; Lane 3: pBD4; Lane 4: pBD114; Lane 5: pBD115; Lane 6: pBD119; Lane 7: pBD123; Lane 8: pBD115; Lane 9: pBD128; Lane 10: pBD129; Lane 11: pBD135; Lane 12: pBD131; Lane 13: pBD130; Lane 14: pEP2C; Lane 15: PG-1; Lane 16: PMAP23; Lane 17: pBD1; Lane 18: pBD2; Lane 19: pBD3.

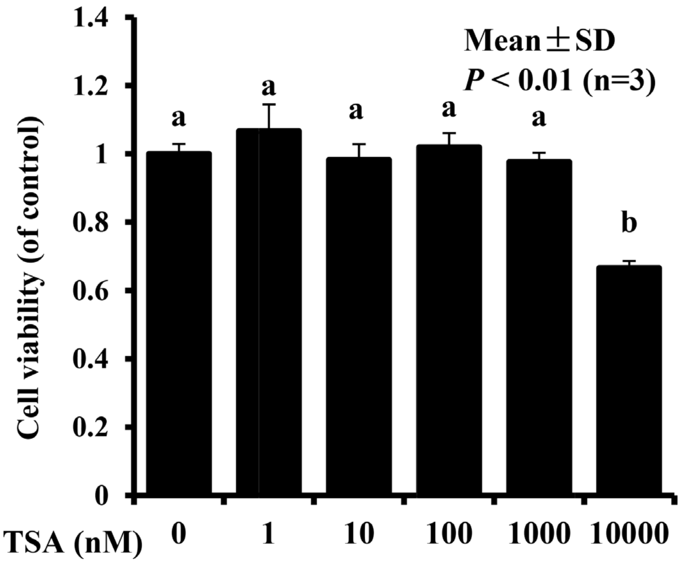

Supplementary Figure 2: Effects of TSA on the viability. PK-15 cells were treated with the indicated concentrations of TSA, ranging from 0–10 μM, for 24 hours. Cell viability was measured using the CCK-8 assay. All CCK-8 values were normalized to the control serial concentrations of TSA for 24 hours.

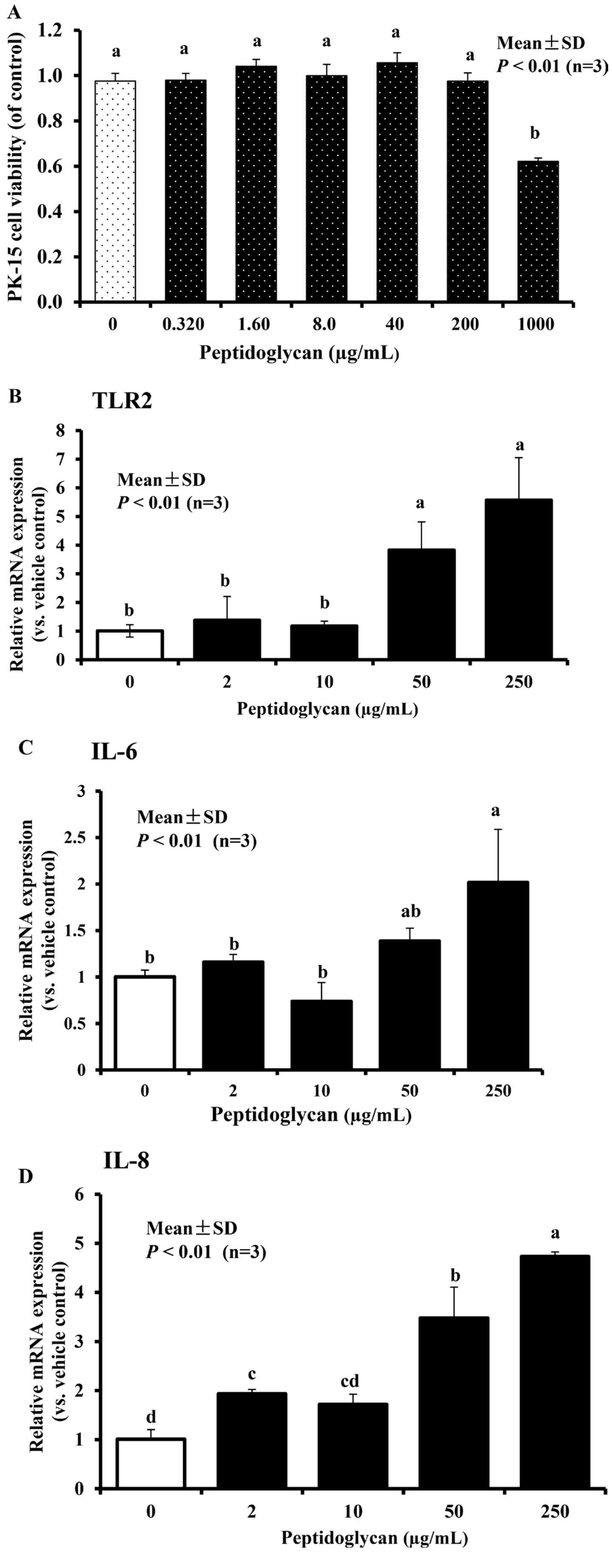
A
PK-15 cell viability (of control)
Mean±SD
P < 0.01 (n=3)
0 0.320 1.60 8.0 40 200 1000
Peptidoglycan (μg/mL)
B
TLR2
Relative mRNA expression (vs. vehicle control)
Mean±SD
P < 0.01 (n=3)
0 2 10 50 250
Peptidoglycan (μg/mL)
C
IL-6
Relative mRNA expression (vs. vehicle control)
Mean±SD
P < 0.01 (n=3)
0 2 10 50 250
Peptidoglycan (μg/mL)
D
IL-8
Relative mRNA expression (vs. vehicle control)
Mean±SD
P < 0.01 (n=3)
0 2 10 50 250
Peptidoglycan (μg/mL)

E

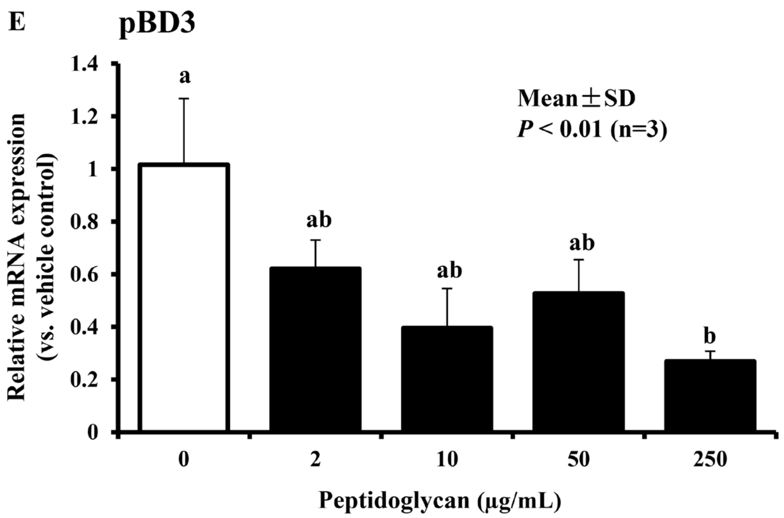

F

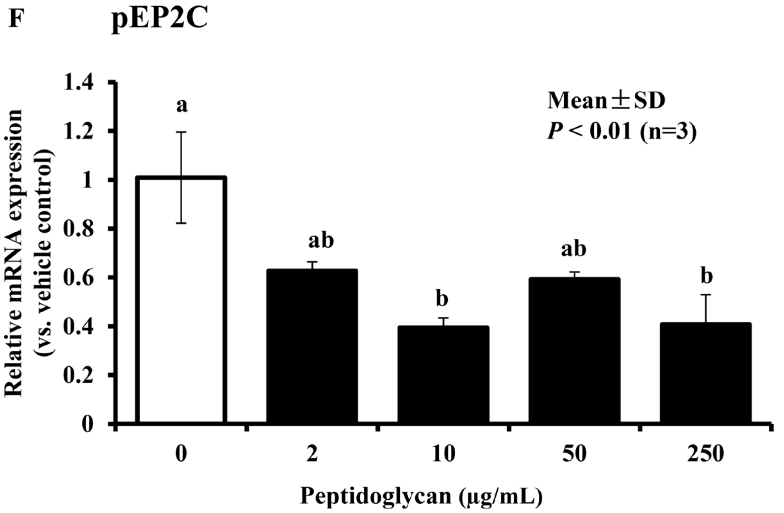

Supplementary Figure 3: Regulation of TLR2, AMP, and cytokines mRNA expression in porcine kidney cells in response to the TLR2 ligand- peptidoglycan. (**A**) PK-15 cells were treated with the indicated concentrations of PGN, ranging from 0–1,000 µg/mL, for 24 hours. Cell viability was measured using the CCK-8 assay. All CCK-8 values were normalized to the control serial concentrations of PGN for 24 hours. (**B**–**F**) TLR2, IL-6, IL-8, pBD3, and pEP2C gene expression were determined by qRT-PCR after treatment with 0, 0.320, 1.60, 8.0, 40, 200, and 1000 µg/mL PGN for 24 hours. Similar results were obtained in repeated experiments (more than two) using different cell preparations. Abbreviations: PGN, peptidoglycan. Means with different letters are significantly different at $P < 0.01$.

Supplementary Table 1: List of primers used for qRT-PCR

Target gene	Sequence (5′–3′)	Reference/accession
pBD-3	Forward: GAAGTCTACAGAAGCCAAAT	b
	Reverse: GGTAACAAATAGCACCATAA	
pEP2C	Forward: GTTGACCTGGGAGCCAAAG	BK005522.1
	Reverse: GCACAGATGACAAAGCCTCA	
pBD115	Forward: CTTAGCTGTCCTTGTGGTCC	a
	Reverse: CAAGCCTTAGCTGTACTTGC	
pBD123	Forward: TGGAATCTTCACGGCAAAT	a
	Reverse: TGATACTTGGGCTTCACACA	
pBD128	Forward: GGTTCTCATTATCCTGCTGT	a
	Reverse: TGTGTTCACTGTGACAGTGG	
NF-κB1 (p50)	Forward: CTCGCACAAGGAGACATGAA	d
	Reverse: ACTCAGCCGGAAGGCATTAT	
NF-κB3 (p65)	Forward: TGTGTAAAGAAGCGGGACCT	KC316023.1
	Reverse: CACTGTCACCTGGAAGCAGA	
IL-1α	Forward: GCT CAA AAC GAA GAC GAA CC	c
	Reverse: TGA TGG TTT TGG GTG TCT CA	
IL-6	Forward: TGGCTACTGCCTTCCCTACC	d
	Reverse: CAGAGATTTTGCCGAGGATG	
IL-8	Forward: CTGGCTGTTGCCTTCTTG	d
	Reverse: TCGTGGAATGCGTATTTATG	
IL-18	Forward: ACTTTACTTTGTAGCTGAAAACGATG	d
	Reverse: T TT AGG TTC AAG CTT GCC AAA	
TLR2	Forward: TCACTTGTCTAACTTATCATCCTCTTG	d
	Reverse: TCAGCGAAGGTGTCATTATTGC	
IL-2	Forward: GGAAGTTAAGAATTACGAGAATGCTG	c
	Reverse: CCTGCTTGGGCATGTAAAAT	
IL-4	Forward: TTGCTGCCCCAGAGAAC	JF906512.1
	Reverse: TGTCAAGTCCGCTCAGG	
IL-10	Forward: CAGATGGGCGACTTGTTG	d
	Reverse: ACAGGGCAGAAATTGATGAC	
IL-12	Forward: TTC CTG TGT CCA TGA AAA CTT	c
	Reverse: AGG TAC CAG TGG CCC TGA AT	
OCLIN	Forward: TCGACTGGATAAAGAGCTGGA	c
	Reverse: TTACTTTTGTAATCCGCAGATCC	
pCLDN4	Forward: CGTACCGACAAGCCCTACTC	NM_001161637.1
	Reverse: CAGTCCAGGGAGAAACCAAG	
pCLDN10	Forward: TGGTTCCATATTTGCCCTGT	NM_001243444.1
	Reverse: CATTGAGCACAGCCCTGAC	
pCLDN15	Forward: GATCTTCGGCTTCTTCATGG	FJ873104.1
	Reverse: GATGGTGTTGGTGGTGATGA	
β–actin	Forward: GGCTCAGAGCAAGAGAGGTATCC	U07786
	Reverse: GGTCTCAAACATGATCTGAGTCATCT	

a [52]; b [53]; c [54]; d [55].
